# Exploring shared pathogenic mechanisms and biomarkers in hepatic fibrosis and inflammatory bowel disease through bioinformatics and machine learning

**DOI:** 10.3389/fimmu.2025.1533246

**Published:** 2025-05-12

**Authors:** Shangkun Li, Haoyu Li, Mingran Qi

**Affiliations:** ^1^ Clinical Medicine, China-Japan Union Hospital of Jilin University, Changchun, Jilin, China; ^2^ Department of Pathogen Biology, College of Basic Medical Sciences, Jilin University, Changchun, Jilin, China

**Keywords:** hepatic fibrosis, inflammatory bowel disease, biomarkers, WGCNA, machine learning algorithms

## Abstract

**Background:**

The coexistence of hepatic fibrosis (HF) and inflammatory bowel disease (IBD) represents a significant clinical concern due to their poorly characterized shared pathogenic mechanisms. Current limitations in identifying common biomarkers for comorbid cases impede early dual diagnosis and therapeutic interventions.

**Methods:**

Differentially expressed genes (DEGs) were screened, followed by Weighted Gene Co-expression Network Analysis (WGCNA) to identify disease-associated modules. The key diagnostic biomarkers were determined via a protein-protein interaction (PPI) network combined with two machine learning algorithms. The logistic regression model was subsequently developed based on these key genes. Immune cell infiltration profiling of both diseases was assessed via the CIBERSORT algorithm. The construction of genes-miRNAs and genes-TFs (Transcription Factors) regulatory networks were based on the NetworkAnalyst website. Potential drug-gene interactions were predicted utilizing the DSigDB database. The expression and distribution of these genes were validated through single-cell sequencing analysis.

**Results:**

A sum of 119 up-regulated genes and 17 down-regulated genes were screened, which were enriched in categories associated with immune cell infiltration and chemotaxis, cytokine regulation, metabolic processes, enzymatic activity, and extracellular matrix deposition, based on enrichment analysis. WGCNA revealed four disease-associated gene modules. Four shared diagnostic genes for both diseases were screened, including MMP2, COL1A2, STAT1, and CXCL1. ROC curve analysis confirmed robust diagnostic performance as AUC > 0.7 for individual genes and AUC > 0.85 for combined model. M1 macrophages were significantly increased in both pathologies of diseases. A total of 462 drugs were predicted targeting these biomarkers in the DSigDB database. The four key diagnostic gene expression patterns across diverse cell subpopulations were visualized by single-cell sequencing analysis.

**Conclusion:**

MMP2, COL1A2, CXCL1, and STAT1 were identified as shared biomarkers for IBD and HF, providing a molecular basis for early diagnosis and precision medicine approaches. It elucidated the similarities between HF and IBD in terms of immunity, metabolism, and fibrosis.

## 1 Introduction

Hepatic fibrosis (HF) arises from an imbalance between synthesis and degradation of the extracellular matrix during chronic liver injury and repair processes, leading to aberrant amass of fibrous liver tissue. Etiologies contain alcoholic liver disease, viral hepatitis (such as hepatitis B and C), and non-alcoholic fatty liver disease (NAFLD) (1). Inflammatory bowel disease (IBD), encompassing ulcerative colitis and Crohn’s disease (CD), is featured with ongoing and recurrent intestinal inflammation. While the precise pathogenesis of IBD remain elusive, they are closely related to environmental, genetic, infectious, and immune factors (2). Prolonged intestinal inflammation can ultimately progress to intestinal fibrosis and stricture formation. Reports indicate that, within a decade of being diagnosed, surgical intervention is necessary for 70% of Crohn’s disease patients, with half of these cases attributed to developed intestinal strictures (3).

As the most widespread chronic diseases affecting the digestive system, their concurrence is not rare. Recent research has shown that 42.00% of IBD patients have metabolic-associated fatty liver disease (MAFLD), with 9.50% progressing to advanced hepatic fibrosis. The study also indicated that IBD independently predicts MAFLD (adjusted odds ratio [aOR], 1.99; P<0.001) and serves as an independent risk factor for advanced hepatic fibrosis (aOR, 5.55; P<0.001) (4). Moreover, primary sclerosing cholangitis is an immune-mediated hepatobiliary disorder pathologically defined by peripheral fibrosis of intrahepatic and extrahepatic bile ducts, progressing to cirrhosis during its advanced stages. It is indicated to be related to IBD, with prevalence rates reaching up to 88% in affected cohorts (5). Furthermore, the use of immunosuppressants, a common therapeutic approach for IBD, may predisposes patients to hepatitis virus reactivation in those co-infected with both IBD and viral hepatitis. This drug-induced hepatotoxicity induces potential liver dysfunction and accelerates hepatic fibrosis progression, as exemplified by anti-TNF-α agents (6, 7). Studies have also reported that advanced hepatic fibrosis, namely cirrhosis, independently negatively impacts the prognosis of hospitalized IBD patients (8). In summary, the co-occurrence of HF and IBD warrants attention, and it holds significant research priority when delving into their convergent mechanisms and potential shared therapeutic targets.

Currently, the diagnostic paradigms of hepatic fibrosis primarily relies on histopathological evaluation of pathological biopsy, along with non-invasive examination methods based on serological or ultrasonic indicators (9, 10). The diagnosis of IBD encompasses serum and fecal testing, intestinal ultrasonography, endoscopic visual inspection, and histopathological confirmation, among others (11–13). However, these methodologies are inadequate to provide accurate diagnoses at the early stages of diseases, with the presence of false positives or false negatives, and they do not offer profound insights into their underlying mechanisms. Identifying shared biomarkers for both diseases could help overcome these limitations, especially in cases where IBD coexists with hepatic fibrosis. Additionally, predicting pharmacological agents that target shared biomarkers may facilitate the treatment of both conditions, thereby enabling dual-purpose therapeutic strategies, minimizing the risk of polypharmacy, and enhancing clinical outcomes for patients with concurrent HF and IBD.

Recent evidence highlights bidirectional gut-liver crosstalk as a mechanistic bridge: impaired hepatic bile acid synthesis in fibrosis modifies the composition of intestinal microbiota, leading to dysbiosis and barrier dysfunction (14–16). Conversely, gut-derived bacterial metabolites and pathobionts translocate via portal circulation, triggering hepatic inflammation through pattern recognition receptors (17, 18). However, existing studies predominantly emphasize phenomenological associations rather than systematic identification of shared molecular drivers. Critical gaps persist in three aspects (1): No integrative analysis of disease-specific transcriptomes to pinpoint overlapping pathways; (2) Lack of shared biomarkers capable of dual diagnosis and therapeutic interventions; (3) Limited exploration of immune-microenvironment interplay across both diseases.

To overcome these gaps, we employed a comprehensive analysis of integrating HF and IBD transcriptomic profiles derived from microarray datasets in the GEO database. Using bioinformatic methods, we screened for differentially expressed genes (DEGs) and identified gene modules common to both diseases. Then, we established a protein-protein interaction (PPI) network and utilized machine learning algorithms to find out the key diagnostic genes. We also explored the correlations between these genes and various immune cells, established genes-miRNAs and genes-TFs regulatory networks, and predicted corresponding drugs for the key diagnostic genes utilizing the DSigDB database. Finally, we verified the expression and distribution of these genes through single-cell data. Overall, our study offers fresh perspectives on comprehending the shared pathogenic mechanisms of HF and IBD, and identifies shared biomarkers for these two disorders.

## 2 Materials and methods

### 2.1 Data collection

We chose three HF-related datasets, with GSE84044 serving as the training set, which includes 43 samples at stage 0 and 28 samples at stages 3-4. The datasets GSE49541 and GSE6764 were used as validation sets. Specifically, GSE49541 comprises 40 samples at stages 1–2 and 32 samples at stages 3-4, while GSE6764 includes 10 normal samples and 13 samples of cirrhosis. These three datasets are all relevant to gene expression profiles of human liver tissue samples through microarray technology, with the platform being GPL570.

For IBD, two datasets were selected. GSE126124 served as the training set, with 57 IBD samples and 21 normal samples. GSE47908 was treated as the validation set, including 39 IBD samples and 15 normal samples. The samples selected from the aforementioned two datasets are both human colon biopsy samples, and the gene expression profiles were detected through microarray technology. The platform for GSE126124 is GPL6244, while the platform for GSE47908 is GPL570.

### 2.2 Screening of DEGs and enrichment analysis

We utilized the “limma” R package to identify DEGs between groups in the GSE84044 and GSE126124 datasets. The criteria for screening DEGs were “|log2 Fold Change (FC) | > 0.5, the adjusted p-value< 0.05”. Subsequently, we plotted volcano plots of DEGs via the “ggplot” R package, with genes satisfying “|log2 FC| ≥ 2 and the adjusted p-value< 0.01” highlighted. Heatmaps were generated via the “pheatmap” R package to display the expression patterns of the top 50 genes with the smallest p-values in each dataset. Venn diagrams were drawn via the ‘VennDiagram’ R package to illustrate the intersection of the up-regulated DEGs between the two datasets, as well as the intersection of the down-regulated DEGs. GO and KEGG enrichment analyses were deployed by the enrichGO and enrichKEGG functions in the “clusterProfiler” R package, respectively, and their visualizations were conducted via the dotplot function of “enrichplot” R package.

### 2.3 WGCNA

We applied the “WGCNA” R package to conduct Weighted Gene Co-expression Network Analysis (WGCNA), which aimed at identifying gene modules that are jointly relevant to hepatic fibrosis and IBD. To facilitate a better analysis, we merged the GSE84044 and GSE126124 datasets and eliminated batch effects via the “Combat” function of “sva” R package. Hierarchical clustering of samples was developed utilizing the “hclust” function from the “stats” R package. Outlier samples were excluded by applying the “cutreeStatic” function from the “WGCNA” R package, with the parameter “cutHeight” set at 65 and parameter “minSize” at 10. The “pickSoftThreshold” function was utilized to identify the appropriate soft threshold for constructing networks. Based on the optimal soft threshold selected, network construction and module identification were carried out using the “blockwiseModules” function, with parameter “minModuleSize” set at 60 and parameter “mergeCutHeight” at 0.25. We deployed the Pearson correlation coefficient to describe the correlations between gene modules and disease traits, which were then visualized using a heatmap. Gene modules that were positively or negatively correlated with both diseases were selected. “Gene significance” was used as a metric to quantify the association between genes and disease phenotypes, while “module membership” represented the correlations between genes and a given module. Additionally, genes from the modules commonly associated with both diseases were extracted and subjected to GO and KEGG enrichment analyses via the “clusterProfiler” R package.

### 2.4 Construction of PPI network and machine learning-based screening of key diagnostic genes

The identified common DEGs were submitted to the STRING website (https://string-db.org/) to establish a PPI network, following which genes without connections were eliminated. These genes were subsequently imported into the Cytoscape software, where the MCC algorithm, embedded within the cytohubba plugin, was applied to select the 30 most prominent genes. Two machine learning algorithms were applied to identify key diagnostic genes. The Random Forest (RF) algorithm was implemented using the “randomForest” R package, while Support Vector Machine- Recursive Feature Elimination (SVM-RFE) algorithm was executed through the “e1071” and “MSVM-RFE” R packages. The genes screened by both algorithms for the two diseases were intersected, and the outcomes were visualized through the “ggvenn” R package. Meanwhile, to further demonstrate the comprehensive diagnostic capabilities of these genes, we developed a logistic regression model from the key genes using the “lrm” function from the R package “rms”, and subsequently developed a nomogram using the “nomogram” function. To assess the diagnostic effectiveness of these key genes and the prognostic model for two diseases, ROC curves were generated across various datasets via the “pROC” R package, with the AUC values serving as indicators. Additionally, box plots illustrating gene expression levels across different groups were obtained utilizing the “ggboxplot” function from the “ggpubr” R package.

### 2.5 Immune infiltration analysis

The CIBERSORT algorithm was deployed to identify the proportions of infiltrated immune cells within the tissues. Violin plots visualized immune cell composition disparities between disease and control groups. The correlations of the obtained key diagnostic genes with immune cells were represented by Pearson correlation coefficients and displayed through a heatmap.

### 2.6 Construction of the genes-miRNAs and genes-TFs regulatory networks, and prediction of candidate drugs

The prediction of regulatory miRNAs and transcription factors (TFs) of the key diagnostic genes were carried out using the TarBase and JASPAR databases, respectively. The regulatory networks of genes-miRNAs and genes-TFs were developed via the NetworkAnalyst website (https://www.networkanalyst.ca/). Key diagnostic genes were input into the Enrichr website (https://maayanlab.cloud/Enrichr/), and the DSigDB database was applied for predicting drugs related to these key diagnostic genes.

### 2.7 Single-cell sequencing data analysis

We downloaded the single-cell sequencing dataset GSE136103 related to liver cirrhosis and selected three liver cirrhosis samples and three normal samples for subsequent analysis. Additionally, the IBD-related single-cell sequencing dataset GSE214695 was downloaded, and three Crohn’s disease samples along with three normal samples were chosen. The “Seurat” R package was used for data processing. Data quality control standards were established as “ 200 < nFeature_RNA < 4000, percent.mt < 10, and percent.HB < 3”. Following this, the data were normalized. We scaled the 2000 most variable genes using the “ScaleData” function and then performed dimensionality reduction using the “RunPCA” function. We utilized the “harmony” R package to eliminate batch effects among samples. Cell clustering was conducted by applying the “FindNeighbors” and “FindClusters” functions, and subsequently visualized using the UMAP plot. Cell annotation was carried out through the “singleR” R package, followed by manual correction. The “FeaturePlot” function was employed for UMAP visualization of key diagnostic genes. The “Dotplot” function was utilized to visualize the proportions of cells expressing key diagnostic genes and their average expression levels across different cell types.

## 3 Results

### 3.1 Acquisition of the common DEGs

**Figure 1:**
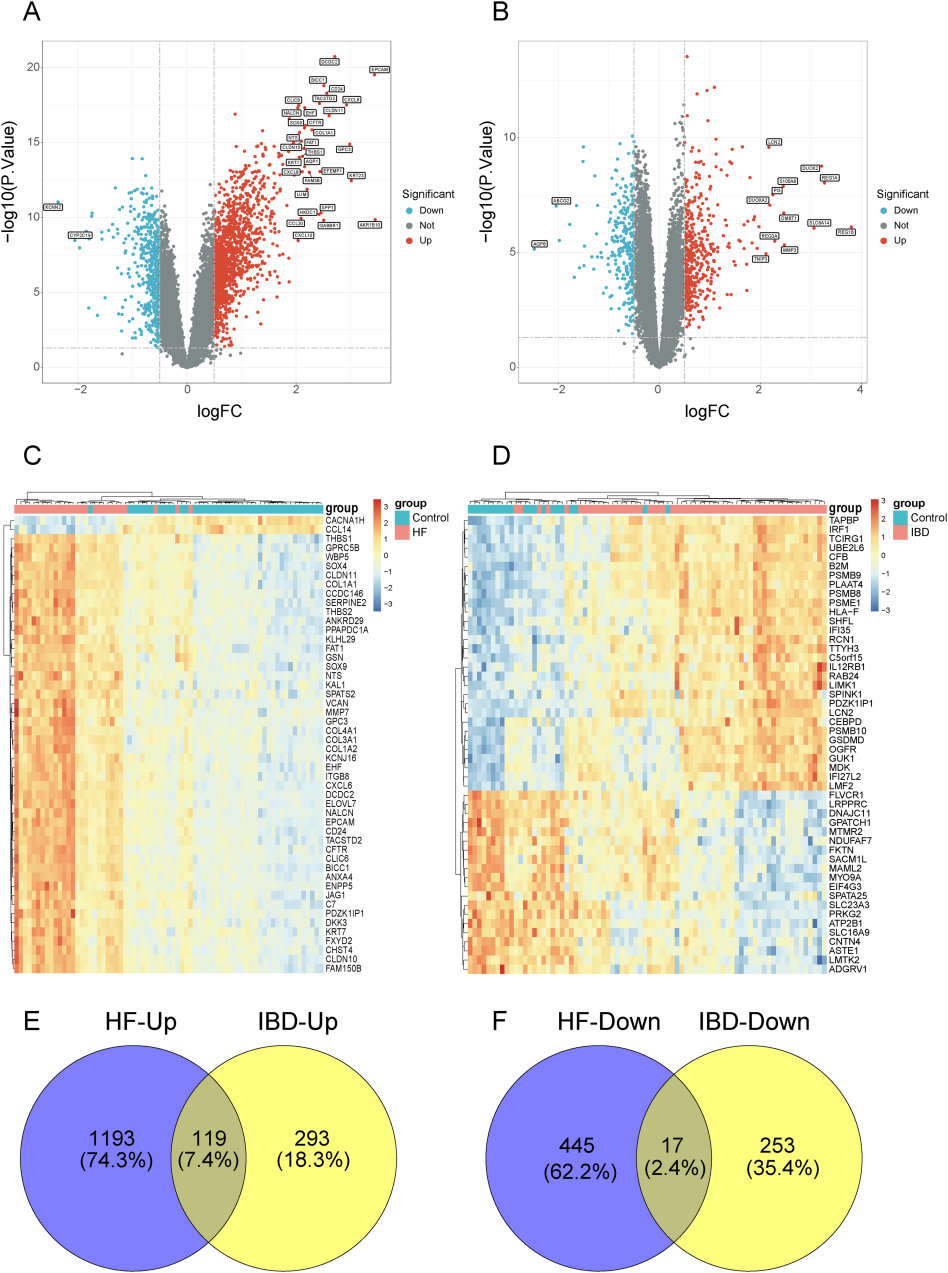
Screening for the common DEGs between GSE84044 and GSE126124. **(A, B)** Volcano plots of the DEGs in GSE84044 and GSE126124. **(C, D)** Heatmaps of the top 50 genes with the most significance in GSE84044 and GSE126124. **(E)** Venn diagram showcasing the intersection of the up-regulated genes between GSE84044 and GSE126124. **(F)** Venn diagram showcasing the intersection of the down-regulated genes between GSE84044 and GSE126124.

As illustrated in the volcano plots, we screened 1312 up-regulated genes and 462 down-regulated genes in GSE84044 dataset (**Figure 1A**), while 412 up-regulated genes and 270 down-regulated genes in GSE126124 dataset (**Figure 1B**). The heatmaps display the expression patterns of top 50 genes with the statistical significance in each dataset, respectively (**Figures 1C, D**). As depicted in the Venn diagram, there are 119 common up-regulated genes and 17 common down-regulated genes between GSE84044 and GSE126124 datasets (**Figures 1E, F**).

### 3.2 Enrichment analysis of the common DEGs

**Figure 2:**
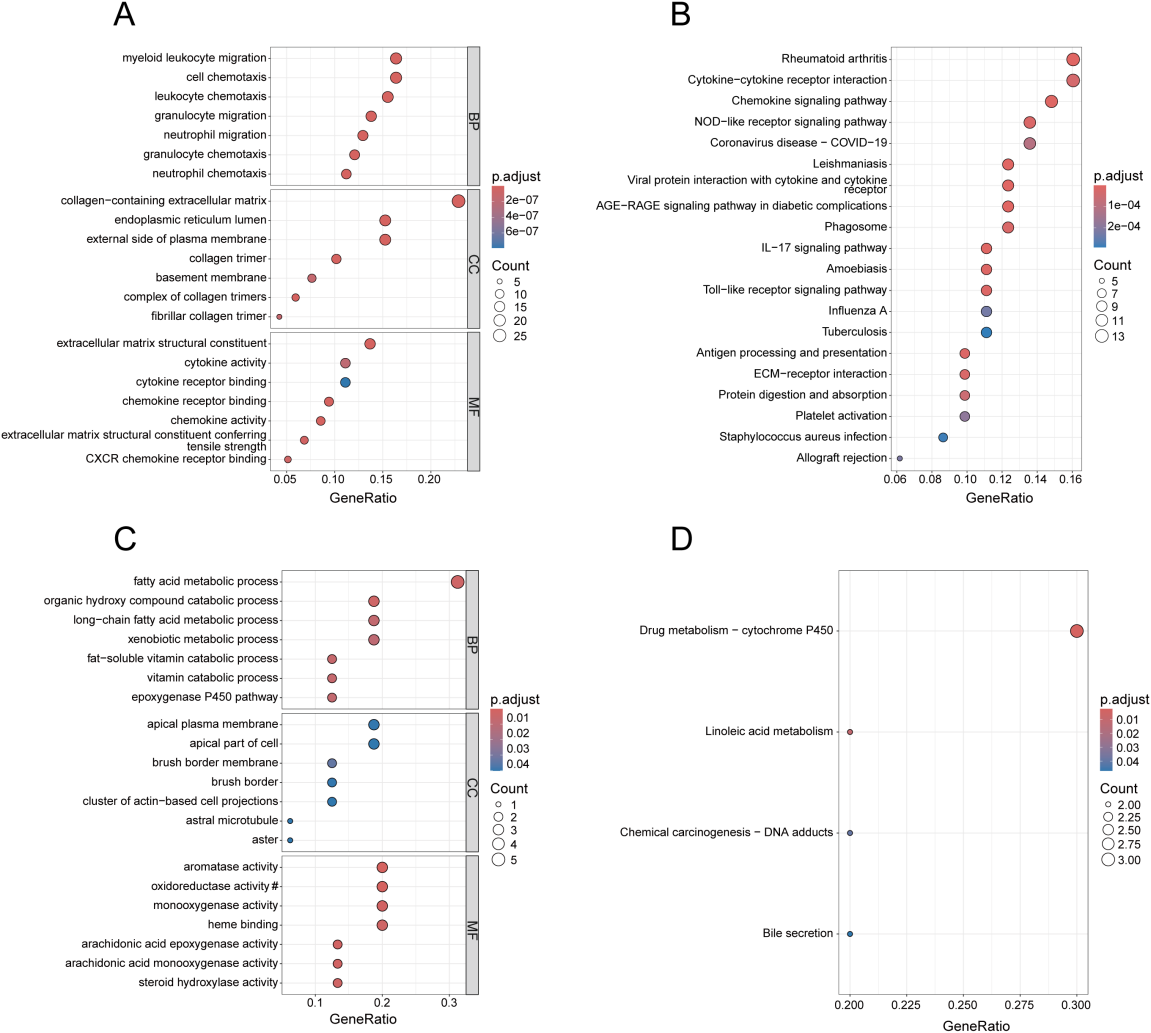
Enrichment analysis of common DEGs. **(A)** GO analysis of the up-regulated genes, presenting the top 7 terms with the smallest p-values in BP, CC and MF. **(B)** KEGG analysis of the up-regulated genes, presenting the top 20 pathways with the most significance. **(C)** GO analysis of the down-regulated genes, presenting the top 7 terms with the most significance in BP, CC and MF. **(D)** KEGG analysis of the down-regulated genes. In **(C)**, the section marked with # has been omitted, and the full term is “oxidoreductase activity, acting on paired donors, involving the incorporation or reduction of molecular oxygen, with reduced flavin or flavoprotein serving as one donor, and incorporating one atom of oxygen”.

In **Figure 2A**, the enrichment results of the common up-regulated genes in BP are mainly associated with immune cell migration and chemotaxis, in CC they mainly relate to extracellular matrix components, and in MF they are predominantly linked to extracellular matrix components and cytokine activity. As shown in **Figure 2B**, the common up-regulated genes are primarily enriched in pathways of Chemokine signaling pathway, Cytokine-cytokine receptor interaction, and NOD-like receptor signaling pathway. Additionally, they encompass pathways associated with other diseases, including Rheumatoid arthritis, Coronavirus disease - COVID-19, and Leishmaniasis. As illustrated in **Figure 2C**, the GO enrichment results of the common down-regulated genes are primarily associated with metabolic processes and enzyme activity. In **Figure 2D**, under the condition of the adjusted p-value < 0.05, the KEGG analysis of the common down-regulated genes enriched in only four terms, with “Drug metabolism-cytochrome P450” being the most significant.

### 3.3 Acquisition of gene modules related to both diseases through WGCNA

**Figure 3:**
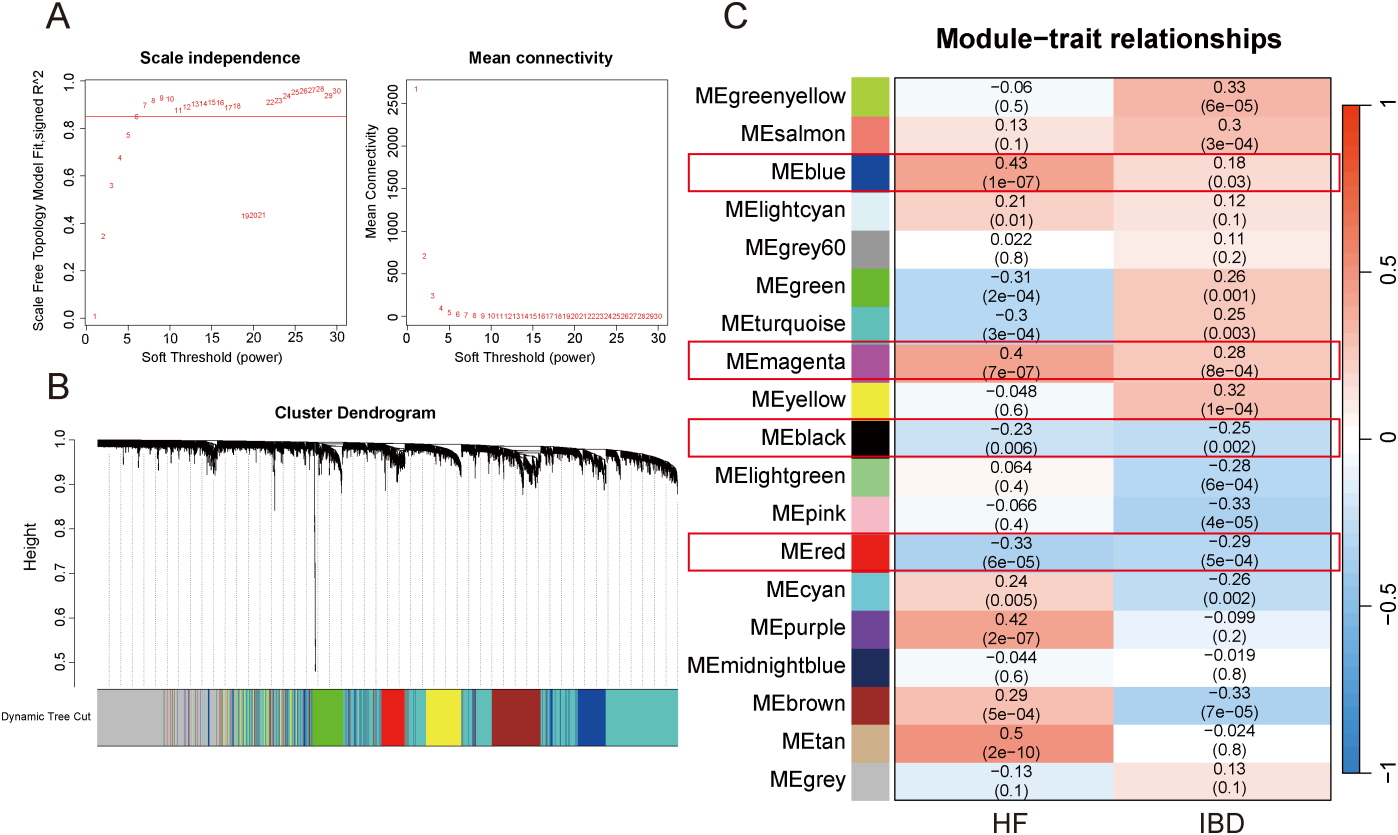
WGCNA. **(A)** Selection of appropriate soft threshold. **(B)** Gene clustering dendrogram. **(C)** Heatmap of correlations between gene modules and diseases.

After merging the GSE84044 and GSE126124 datasets and addressing batch effects, we employed the “cutreeStatic” function with a threshold set at 65 to exclude six outlier samples, retaining a total of 143 samples (**Supplementary Figure 1**; **Figure 2**). Taking into account both the scale-free topology fit index and the average connectivity, the optimal soft-thresholding value (β) was 7 (**Figure 3A**). As depicted in the “cluster dendrogram”, a sum of 18 gene modules were determined (**Figure 3B**). The heatmap displayed the correlation coefficients and corresponding p-values between each gene module and disease traits (**Figure 3C**). Notably, the blue module exhibited positive correlations with both diseases (HF: r=0.18, p=0.03; IBD: r=0.43, p=1e-07), and the magenta module also exhibited a positive relationship (HF: r=0.28, p=8e-04; IBD: r=0.4, p=7e-07). Conversely, the red module was negatively associated with both diseases (HF: r=-0.33, p=6e-05; IBD: r=-0.29, p=5e-04), consistent with the black module (HF: r=-0.23, p=0.006; IBD: r=-0.25, p=0.002). The genes within these modules were considered to have close associations with both diseases. Furthermore, we investigated the association between “Gene Significance” and “Module Membership” of the genes in these modules. As revealed in the scatter plots, all exhibited significantly positive correlations with statistical difference (**Supplementary Figure 3**).

### 3.4 Enrichment analysis of gene modules jointly associated with both diseases

**Figure 4:**
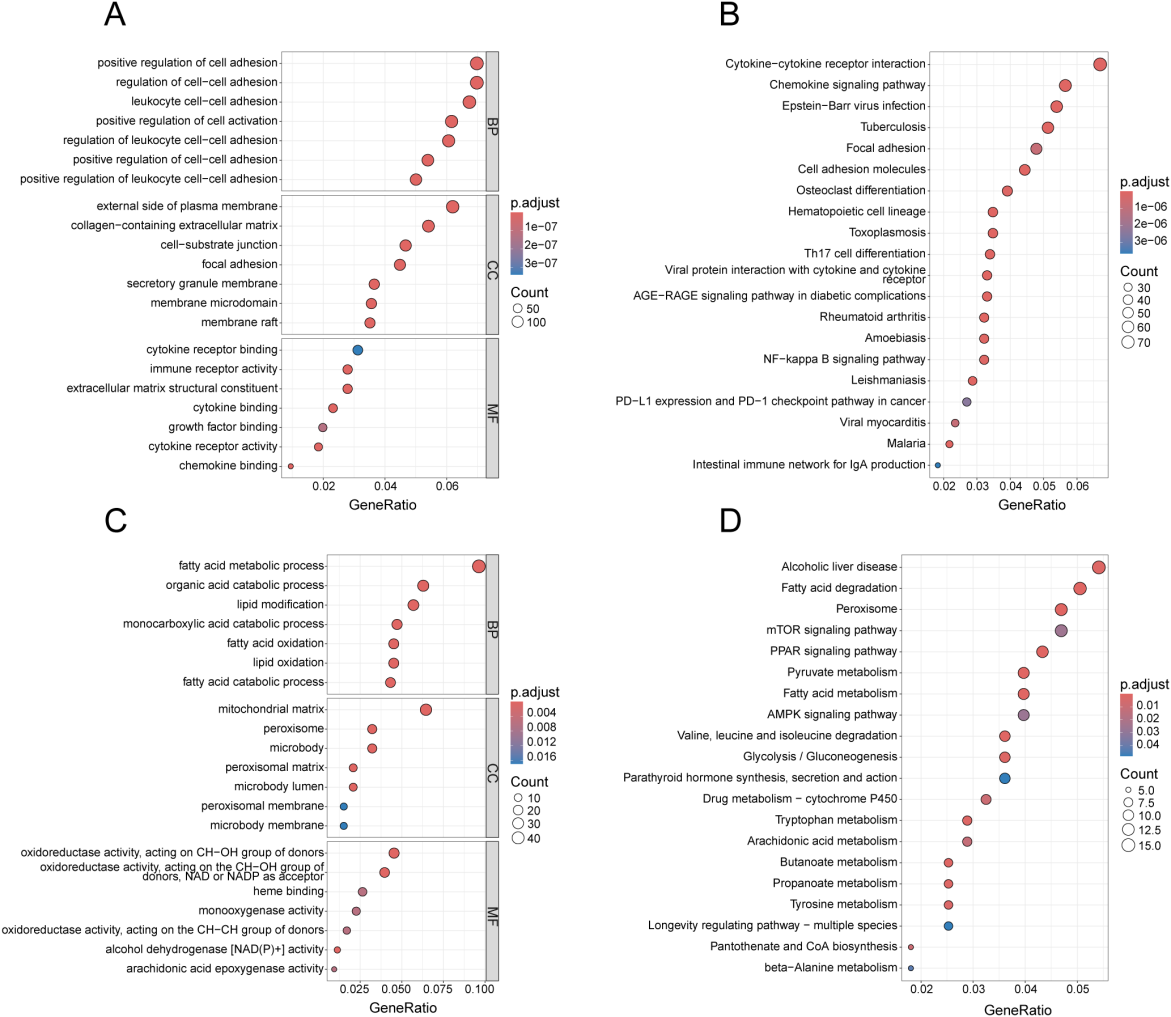
Enrichment analysis of gene modules related to both diseases. **(A)** GO analysis of the blue gene module, presenting the top 7 terms with most significance in BP, CC and MF. **(B)** KEGG analysis of the blue gene module, presenting the top 20 pathways with the most significance. **(C)** GO analysis of the red gene module, presenting the top 7 terms with the smallest p-values in BP, CC and MF. **(D)** KEGG analysis of the red gene module, presenting the top 20 pathways with the most significance.

To identify the underlying biological functions of these gene modules, we applied the genes for GO and KEGG analyses. In **Figure 4A**, the blue gene module is primarily enriched in cell adhesion-related terms in BP, terms associated with cell membrane structure and extracellular matrix components in CC, and terms related to cytokine receptor binding in MF. As illustrated in **Figure 4B**, the KEGG enrichment analysis results for the blue gene module encompass pathways such as Chemokine signaling pathway, Cytokine-cytokine receptor interaction, and Cell adhesion molecules. Additionally, it includes some pathways associated with other diseases, such as Epstein-Barr virus infection, Tuberculosis, and Toxoplasmosis. In **Figure 4C**, the red gene module predominantly enriches in terms associated with metabolic processes in BP, terms related to mitochondria, peroxisomes, and microsomes in CC, and terms linked to enzyme activity in MF. As demonstrated in **Figure 4D**, the KEGG enrichment analysis results of the red gene module still include several metabolism-related pathways. However, both the magenta and black gene modules, under the condition of the adjusted p-value < 0.05, enrich only a few terms (**Supplementary Tables 1**-**3**), and no pathways were found for KEGG enrichment analysis of black gene modules.

### 3.5 Construction of PPI network for the common DEGs

**Figure 5:**
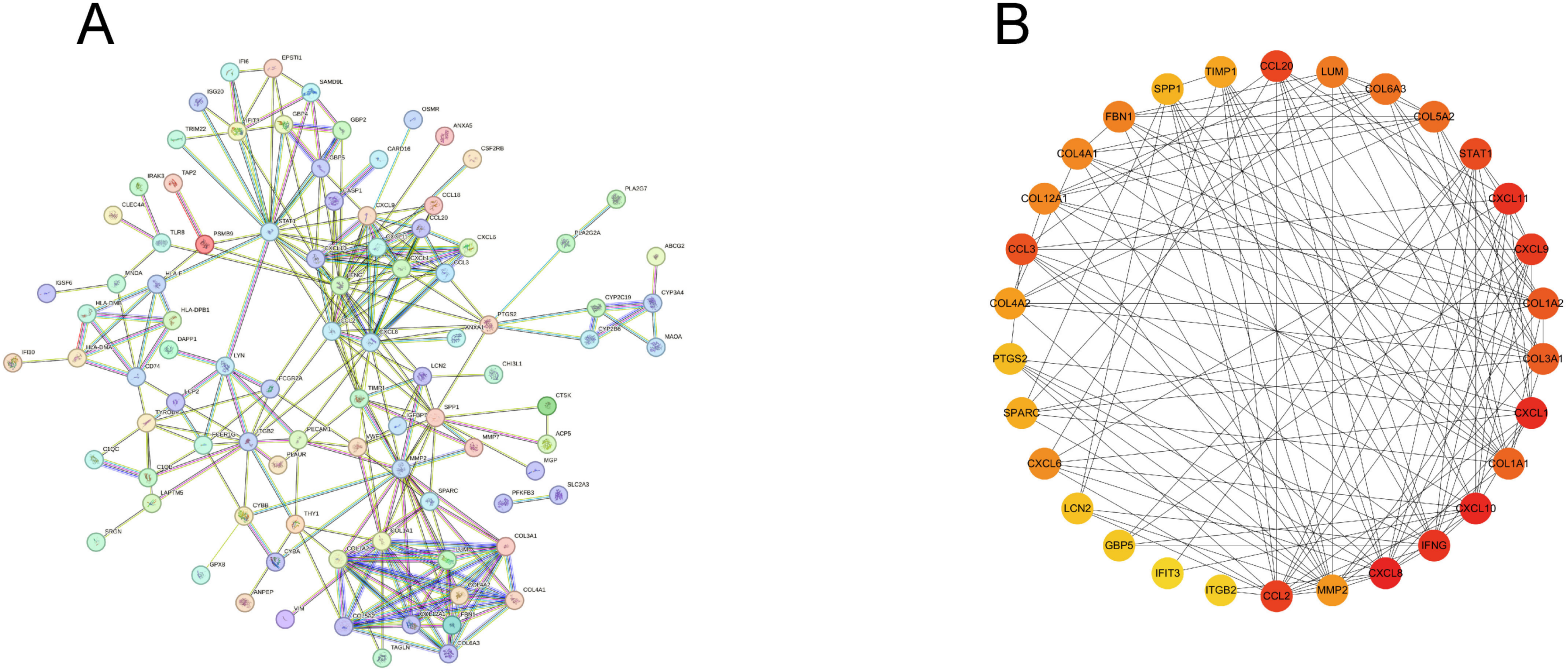
Construction of PPI network. **(A)** PPI network of common DEGs, excluding genes without connections. **(B)** Top 30 genes obtained by MCC algorithm.

To identify key diagnostic genes for both diseases, we input 136 DEGs into the STRING website and removed the nodes without connections to establish a PPI network (**Figure 5A**). Further, we utilized the MCC algorithm in Cytoscape to select the top 30 genes, which served as candidate genes for identifying key diagnostic genes (**Figure 5B**).

### 3.6 Screening of key diagnostic genes via machine learning algorithms

**Figure 6:**
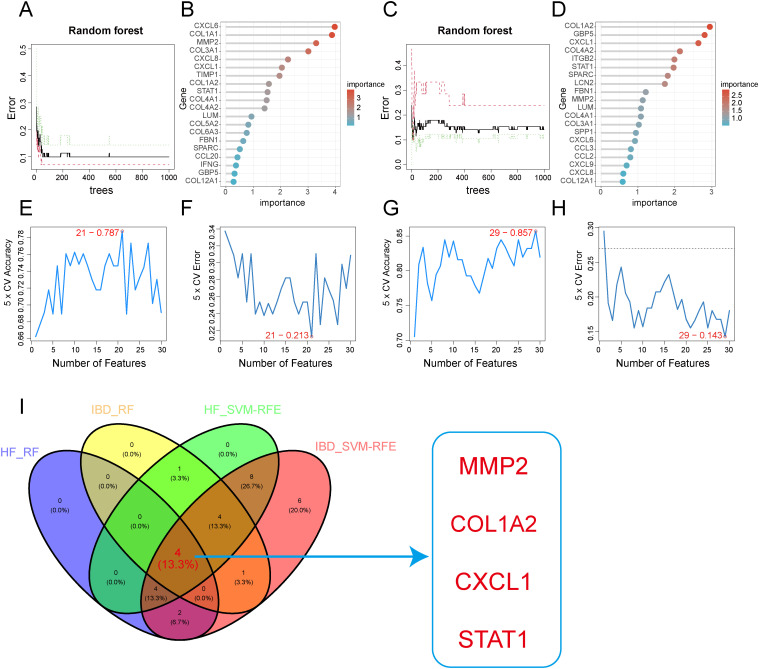
Feature gene selection using machine learning algorithms. **(A, B)** Feature gene selection in GSE84044 using Random Forest(RF). **(C, D)** Feature gene selection in GSE126124 using Random Forest(RF). **(E, F)** Feature gene selection in GSE84044 using SVM-RFE. **(G, H)** Feature gene selection in GSE126124 using SVM-RFE. **(I)** Intersection of feature genes selected by both machine learning algorithms.

**Figure 7:**
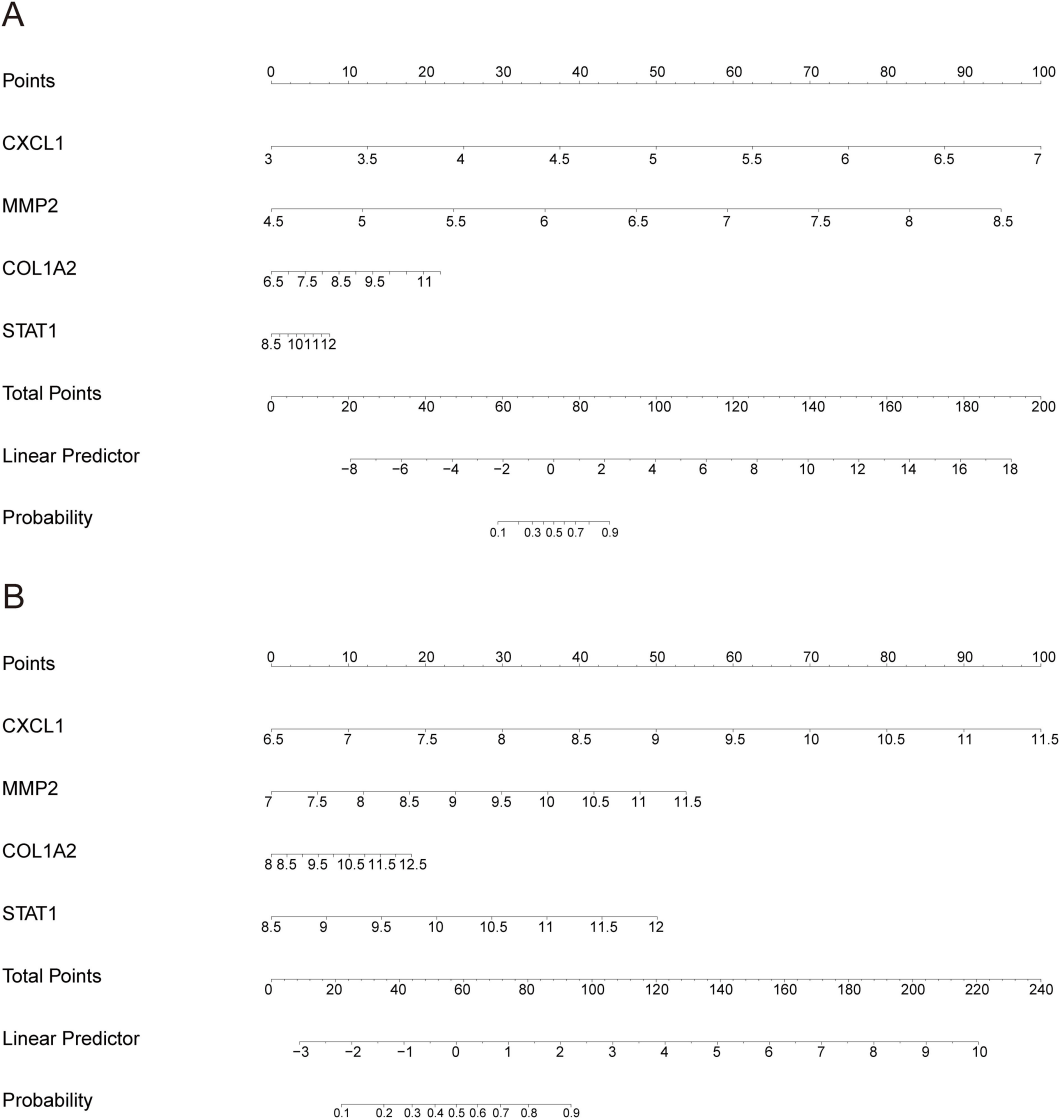
Nomogram of the model: **(A)** Nomogram for the HF training dataset GSE84044; **(B)** Nomogram for the IBD training dataset GSE126124.

Subsequently, we employed two algorithms to select diagnostic genes. The RF algorithm identified the top 10 genes in GSE84044 and GSE126124, with a gene importance scale presenting the top 20 genes (**Figures 6A–D**). The SVM-RFE algorithm selected 21 genes with the lowest 5-fold cross-validation (CV) error and the best 5-fold CV accuracy in GSE84044 (**Figures 6E, F**), and 29 genes with the same criteria in GSE126124 (**Figures 6G, H**). By considering the intersection of these genes, we finally obtained four genes MMP2, COL1A2, STAT1, and CXCL1 (**Figure 6I**). They are considered to have the most diagnostic performance for HF and IBD. To further verify the comprehensive diagnostic capability, we constructed logistic regression models using key diagnostic genes in the training sets. The model formula established in the GSE84044 was expressed as (3.784*CXCL1 + 3.588*MMP2 + 0.665*COL1A2 + 0.327*STAT1), while the model developed in the GSE126124 was formulated as (1.228*CXCL1 + 0.735*MMP2 + 0.248*COL1A2 + 0.879*STAT1). **Figure 7** presents the nomograms of the models.

### 3.7 Validation of diagnostic efficacy of key diagnostic genes and the model

**Figure 8:**
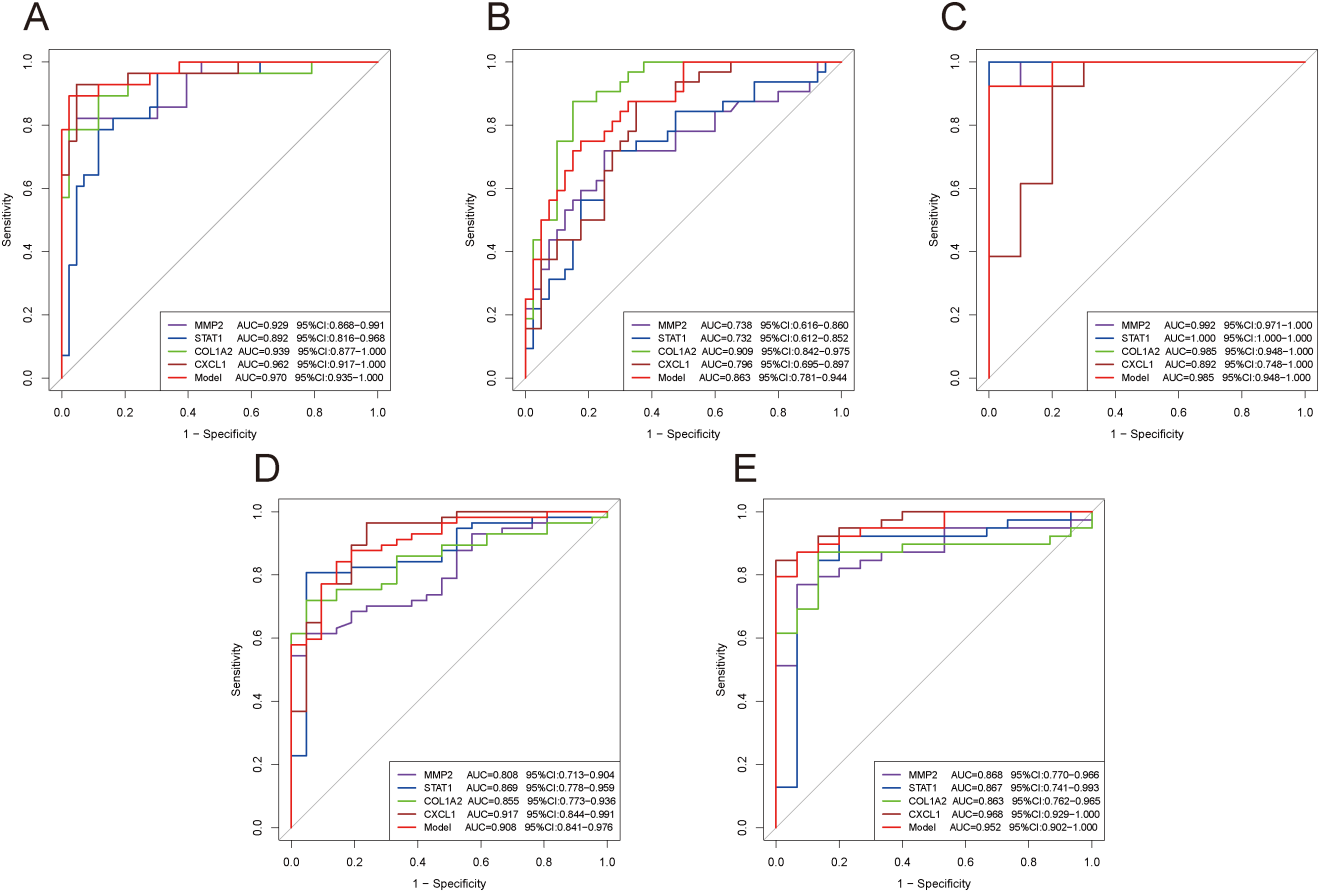
ROC curves evaluating the diagnostic efficacy of key genes and the model. **(A–E)** ROC curves of four genes and the model in different datasets: GSE84044 **(A)**, GSE49541 **(B)**, GSE6764 **(C)**, GSE126124 **(D)**, and GSE47908 **(E)**.

We subsequently validated the diagnostic performance of these four genes and the model across different datasets using ROC curves. The results confirmed that the AUC values were greater than 0.7 in each dataset (**Figure 8**), demonstrating excellent diagnostic efficacy of these four genes for both diseases. The model exhibited good diagnostic performance with AUC values above 0.85 in both training and validation sets (**Figure 8**).

### 3.8 Validation of expression levels of key diagnostic genes

**Figure 9:**
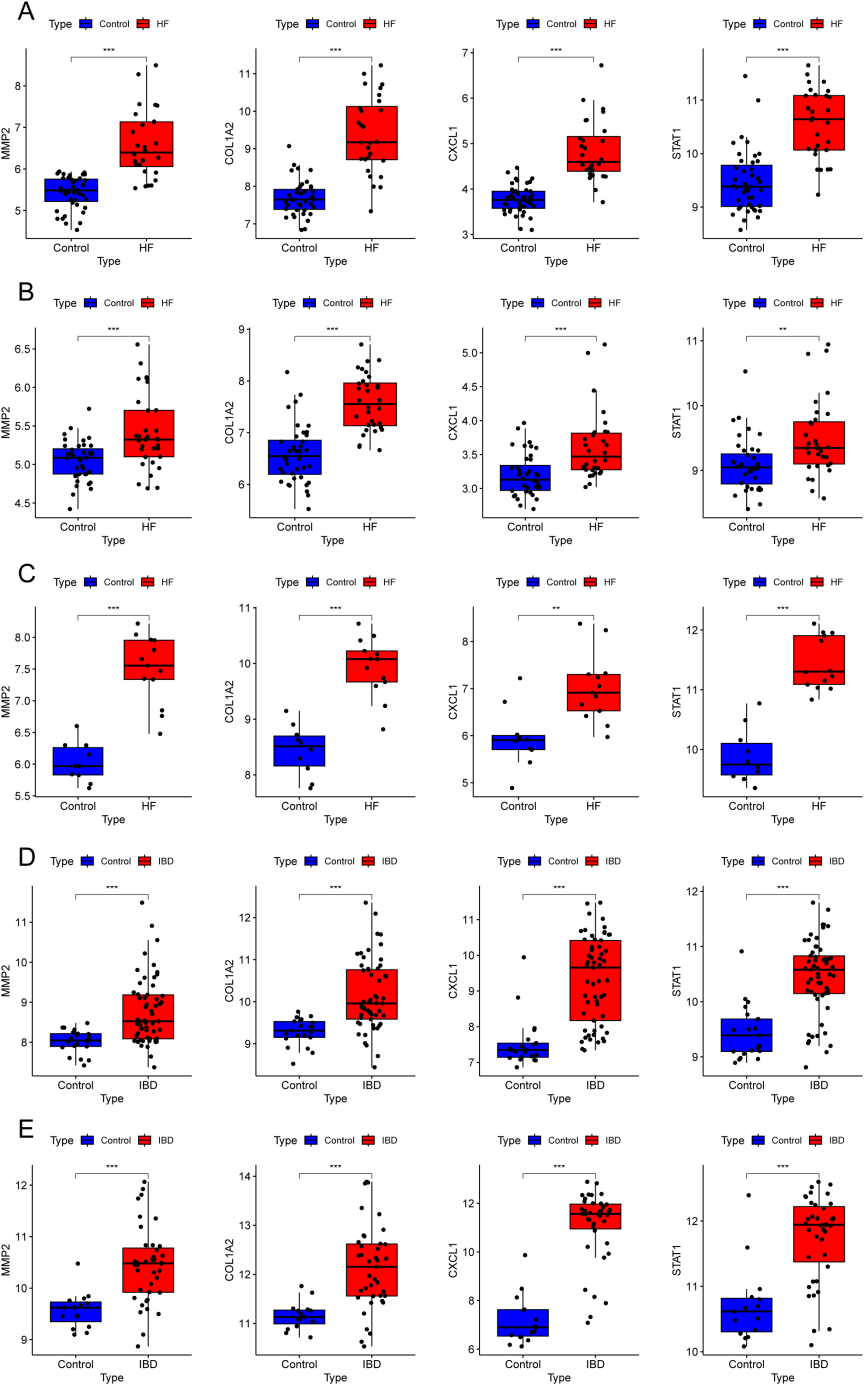
Expression levels of key diagnostic genes in different datasets. **(A–E)** Box plots showing four gene expressions in different datasets: GSE84044 **(A)**, GSE49541 **(B)**, GSE6764 **(C)**, GSE126124 **(D)**, and GSE47908 **(E)**. **P < 0.01, ***P < 0.001.

We visualized the alterations in the four gene expression levels in the GEO database. The findings revealed that the expression levels of these four genes exhibited a consistently upward trend in the disease groups compared to the control groups across all datasets for both diseases (**Figure 9**).

### 3.9 Immune infiltration analysis

**Figure 10:**
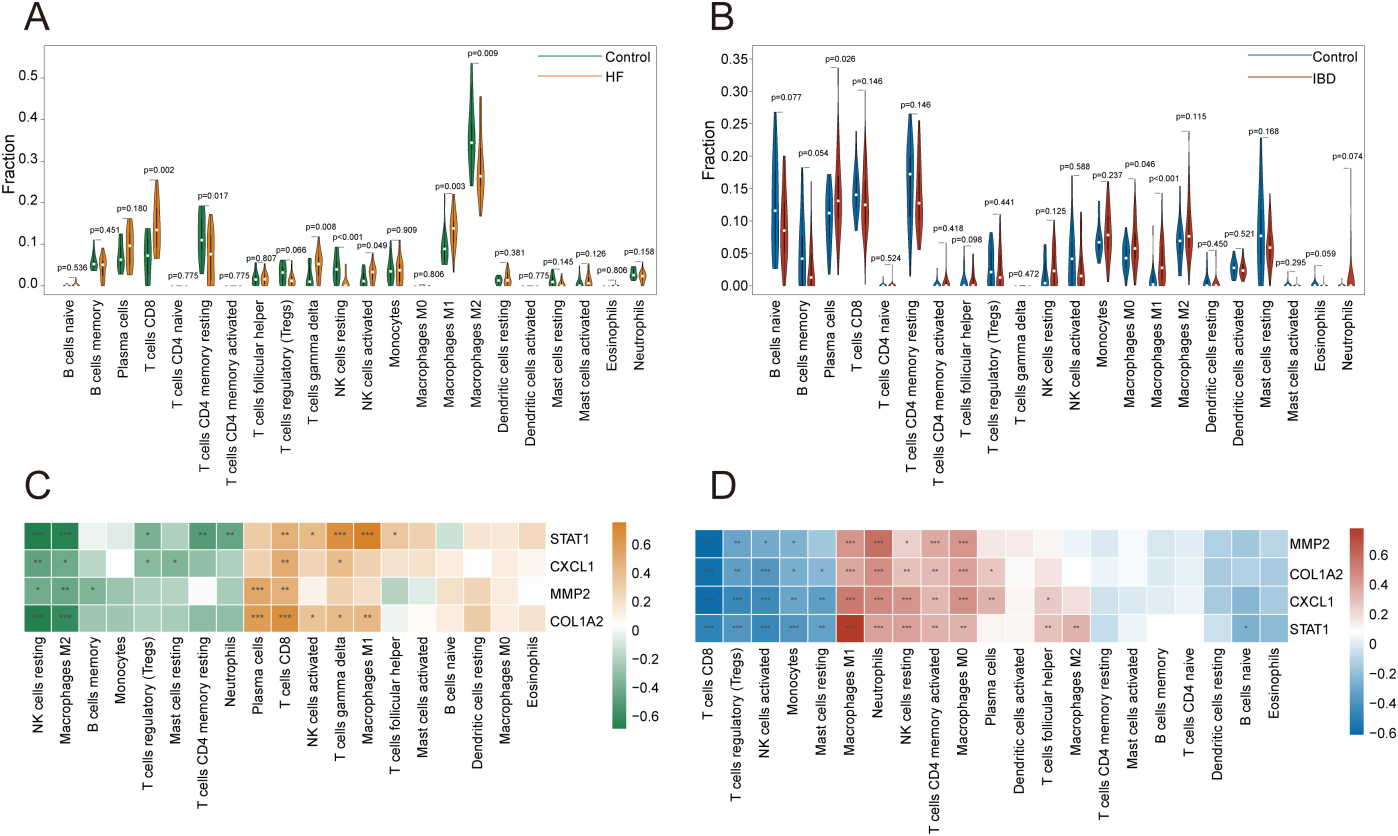
Immune infiltration analysis. **(A, B)** Violin plots of immune cell proportions in GSE84044 and GSE126124. **(C, D)** Heatmap of correlations between key diagnostic genes and immune cells in GSE84044 and GSE126124. *P < 0.05, **P < 0.01, ***P < 0.001.

According to **Figure 10A**, with p-value < 0.05, in the hepatic fibrosis dataset GSE84044, activated NK cells, CD8+ T cells, γδT cells, and M1 macrophages were significantly increased in the disease group, while resting NK cells, resting memory CD4+ T cells, and M2 macrophages were significantly decreased. In the IBD dataset GSE126124, plasma cells, M0 macrophages, and M1 macrophages were significantly elevated in the disease group (**Figure 10B**). The investigation into gene-immune cell relationships in GSE84044 unveiled that the four genes were significantly inversely linked with resting NK cells, M2 macrophages, while positively linked with CD8+ T cells (**Figure 10C**). The genes STAT1, CXCL1, and COL1A2 were significantly positively linked with γδT cells (**Figure 10C**). In GSE126124, the four genes were significantly inversely associated with CD8+ T cells, regulatory T cells (Tregs), activated NK cells, and monocytes, while positively associated with activated memory CD4+ T cells, resting NK cells, neutrophils, M1 macrophages, and M0 macrophages (**Figure 10D**). Additionally, COL1A2, CXCL1, and STAT1 were significantly inversely linked to resting mast cells (**Figure 10D**).

### 3.10 Construction of regulatory networks for genes-miRNAs and genes-TFs

**Figure 11:**
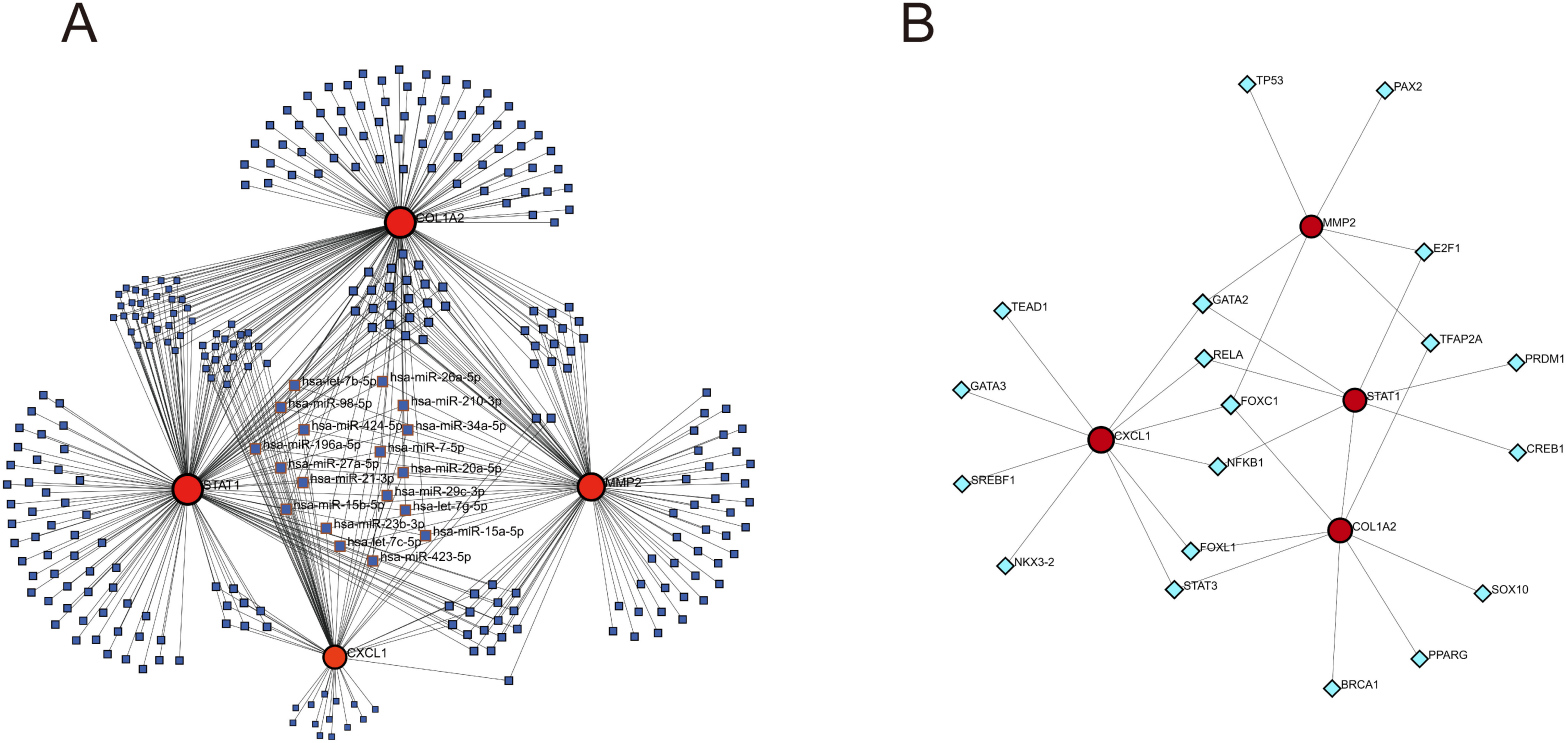
Construction of genes-miRNAs and genes-TFs regulatory networks. **(A)** genes-miRNAs regulatory network. **(B)** genes-TFs regulatory network. The dark blue squares represent miRNAs, with the names of those that have regulatory relationships with all four key diagnostic genes labeled; the red circles represent key genes; the light blue diamonds represent transcription factors.

In the TarBase database, 172 regulatory miRNAs were predicted for the COL1A2, 165 for STAT1, 112 for the MMP2, and 65 for CXCL1. The genes-miRNAs regulatory network comprises a sum of 306 nodes and 514 edges, with 18 miRNAs exhibiting regulatory effects on four genes (**Figure 11A**; **Supplementary Table 4**). In the JASPAR database, 10 TFs were predicted for CXCL1, 8 for COL1A2, 7 for STAT1, and 6 for MMP2. The genes-TFs regulatory network encompasses 23 nodes and 30 edges, where GATA2 acts on MMP2, CXCL1, and STAT1, while FOXC1 influences MMP2, CXCL1, and COL1A2 (**Figure 11B**; **Supplementary Table 5**).

### 3.11 Prediction of drugs related to key diagnostic genes

In the DSigDB database, 462 drugs were predicted with the adjusted p-value<0.05 (**Supplementary Table 6**). The top 10 drugs with the smallest p-values include: “N-Acetyl-L-cysteine CTD 00005305”, “Dinoprostone BOSS”, “PD 98059 CTD 00003206”, “Arsenious acid CTD 00000922”, “Phenylarsine oxide CTD 00001378”, “Acetovanillone CTD 00002374”, “22-Hydroxycholesterol CTD 00000121”, “alpha-Neu5Ac BOSS”, “Cyclohexanecarboxamide, 4-(1-aminoethyl)-N-4-pyridinyl-, trans- CTD 00003513”, and “Electrocorundum CTD 00005364” (**Table 1**).

**Table 1:** Top 10 Drugs ranked by the p-values in the DSigDB database.

Term	p-value	Adjusted p-value	Genes
N-Acetyl-L-cysteine CTD 00005305	6.04E-08	3.38E-05	COL1A2;STAT1;MMP2;CXCL1
Dinoprostone BOSS	3.68E-06	0.001028778	STAT1;MMP2;CXCL1
PD 98059 CTD 00003206	8.80E-06	0.001640489	COL1A2;STAT1;MMP2
Arsenenous acid CTD 00000922	1.69E-05	0.002108219	COL1A2;STAT1;MMP2;CXCL1
phenylarsine oxide CTD 00001378	1.89E-05	0.002108219	STAT1;MMP2
Acetovanillone CTD 00002374	2.83E-05	0.002259251	STAT1;CXCL1
22-Hydroxycholesterol CTD 00000121	2.96E-05	0.002259251	STAT1;CXCL1
alpha-Neu5Ac BOSS	3.23E-05	0.002259251	COL1A2;MMP2
Cyclohexanecarboxamide, 4-(1-aminoethyl)-N-4-pyridinyl-,trans- CTD 00003513	4.12E-05	0.002558954	COL1A2;MMP2
Electrocorundum CTD 00005364	5.47E-05	0.003056863	COL1A2;MMP2

### 3.12 Validation of expression and distribution of key diagnostic genes in single-cell sequencing data

**Figure 12:**
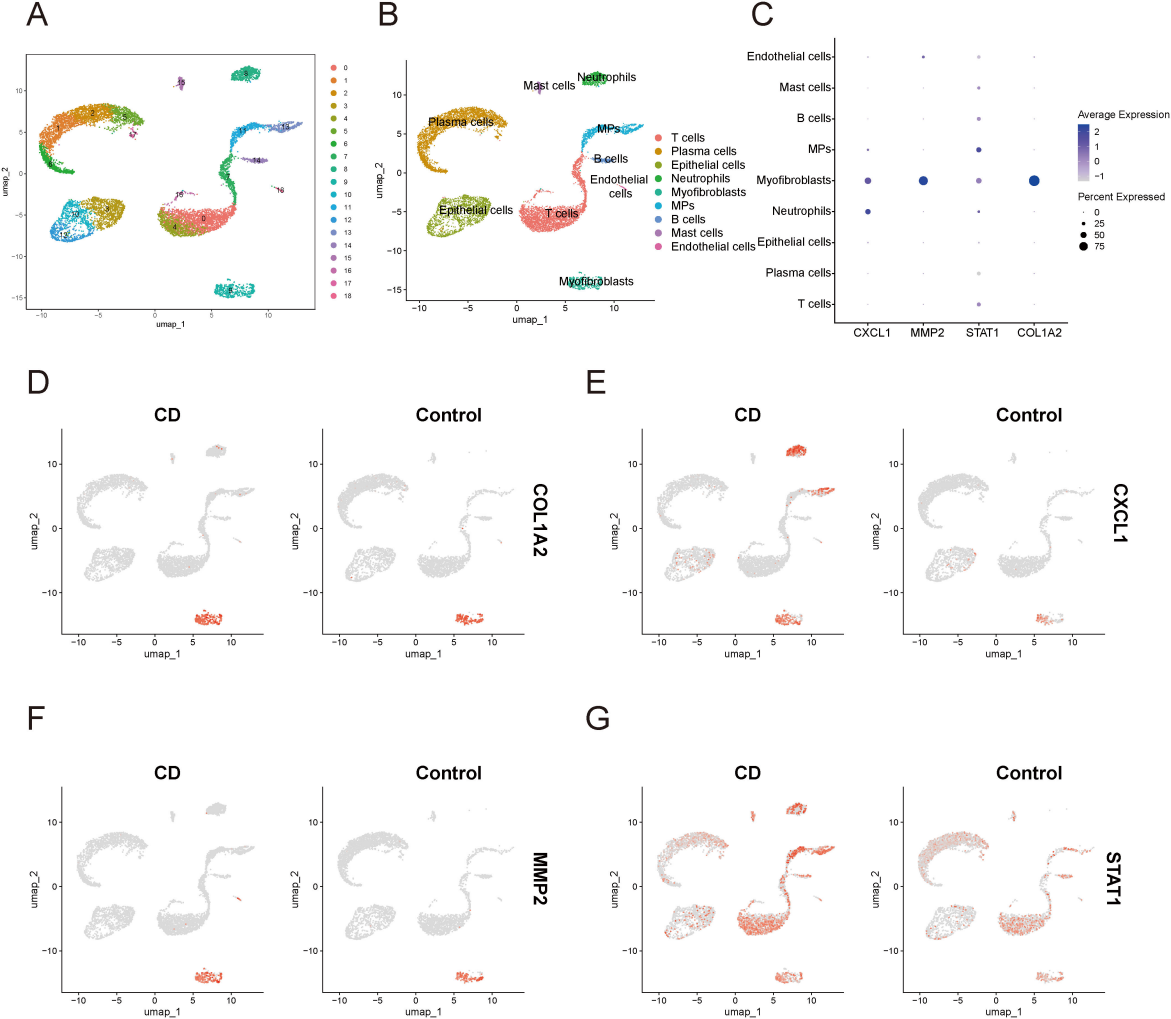
Single-cell sequencing analysis related to IBD **(A)** UMAP plot of cell clustering. **(B)** UMAP plot of different cell populations after cell annotation. **(C)** Bubble plot showing expression levels and proportions of key diagnostic genes in different cell populations. **(D–G)** UMAP plot of expression and distribution of key diagnostic genes across different cell populations: COL1A2 **(D)**, CXCL1 **(E)**, MMP2 **(F)**, and STAT1 **(G)**. MPs, mononuclear phagocytes; CD, Crohn’s disease.

In the single-cell data of IBD, all cells were partitioned into 18 clusters (**Figure 12A**), and through cell annotation, a total of 9 distinct cell populations were obtained: T cells, plasma cells, epithelial cells, neutrophils, myofibroblasts, mononuclear phagocytes, B cells, mast cells, and endothelial cells (**Figure 12B**). Detailed annotation information is provided in the **Supplementary Materials** (**Supplementary Figure 4**). The bubble plot (**Figure 12C**) revealed that MMP2 and COL1A2 were primarily expressed in myofibroblasts, while STAT1 exhibited a certain level of expression across all cell types, with particularly high expression in myofibroblasts, and mononuclear phagocytes. Additionally, the expression of CXCL1 was mainly elevated in myofibroblasts and Neutrophils. The UMAP plot (**Figures 12D–G**) illustrated the expression and distribution of these four genes among different cell populations.

**Figure 13:**
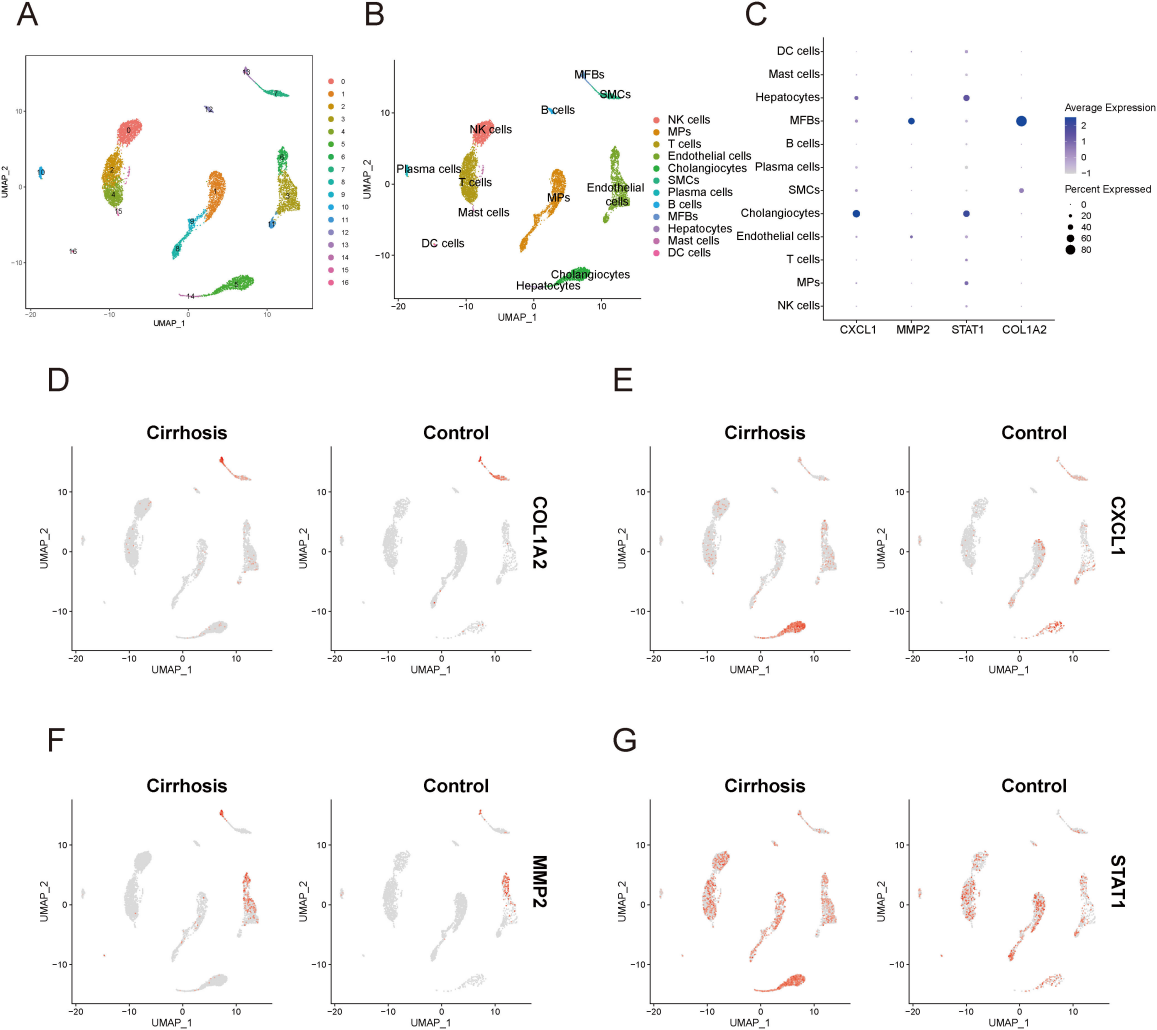
Single-cell sequencing analysis related to cirrhosis. **(A)** UMAP plot of cell clustering. **(B)** UMAP plot of different cell populations after cell annotation. **(C)** Bubble plot showing expression levels and proportions of key diagnostic genes in different cell populations. **(D–G)** UMAP plot of expression and distribution of key diagnostic genes across different cell populations: COL1A2 **(D)**, CXCL1 **(E)**, MMP2 **(F)**, and STAT1 **(G)**. MPs, mononuclear phagocytes; DC cells, dendritic cells; MFBs, myofibroblasts; SMCs, smooth muscle cells.

In the single-cell data of HF, all cells were categorized into 16 clusters (**Figure 13A**). Subsequent cellular annotation revealed 12 cell populations: NK cells, mononuclear phagocytes, T cells, endothelial cells, cholangiocytes, smooth muscle cells, plasma cells, B cells, myofibroblasts, hepatocytes, mast cells, and dendritic cells (**Figure 13B**). Comprehensive annotation details are available in the **Supplementary Materials** (**Supplementary Figure 5**). The bubble plot (**Figure 13C**) showed that MMP2 and COL1A2 was primarily expressed in myofibroblasts. STAT1 was expressed in almost all cell populations, with significantly high expression in hepatocytes and cholangiocytes. CXCL1 was primarily highly expressed in Hepatocytes and cholangiocytes. The UMAP plot (**Figures 13D–G**) illustrated the expression and distribution of these four genes among different cell populations.

## 4 Discussion

A growing body of research has indicated a high prevalence of coexisting HF and IBD, which complicates the management and treatment of these conditions. Furthermore, these two diseases interact through the gut-liver axis and exhibit numerous similarities in their pathological mechanisms. To investigate the common pathogenic mechanisms underlying both diseases and to identify shared biomarkers, we conducted a series of analyses utilizing cutting-edge bioinformatic methods.

We screened for common DEGs between the two diseases and identified gene modules that were correlated with both diseases through WGCNA analysis. Further analysis was employed to elucidate the functional enrichment of the key genes. The results indicated that terms related to the extracellular matrix, such as “collagen-containing extracellular matrix” were significantly enriched among the up-regulated genes. The amass of extracellular matrix is a hallmark of fibrosis. Hepatic stellate cells (HSCs) are integral to the pathogenesis of HF, where they can transform into myofibroblasts under the influence of profibrotic factors, releasing large amounts of collagen and extracellular matrix (ECM) degradation inhibitors, thereby promoting ECM deposition (19). Intestinal fibrosis represents a pathologically significant sequela of IBD, where mesenchymal cells, upon stimulation by profibrotic factors, can differentiate into activated myofibroblasts, which subsequently generate substantial amounts of ECM, thereby promoting the development of fibrosis (20). In addition, pro-fibrotic factors such as TGF-β, PDGF, and IGF-I, which can be produced by multiple cell types, are capable of effectively activating myofibroblasts (20). These studies are consistent with our findings, which further confirm that the fibrotic process is a common feature of both HF and IBD.

**Figure 14:**
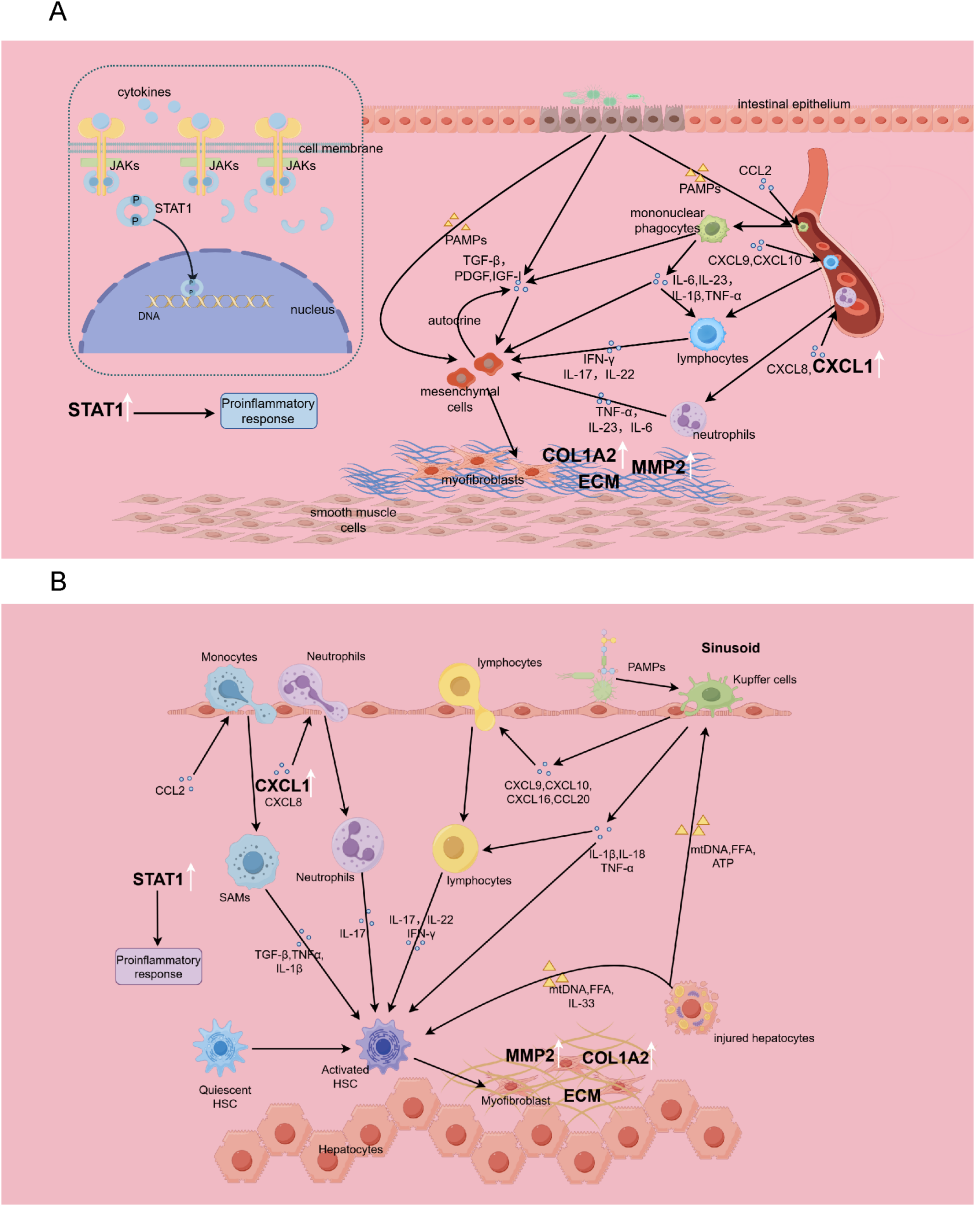
The immune-fibrosis regulatory network in IBD **(A)** and HF **(B)**, and the role of biomarkers therein. This picture is drawn by Figdraw. SAMs, scar-associated macrophages.

Our analysis further revealed marked enrichment of cytokine activity regulation within the up-regulated genes, underscoring that there are many common cytokines and immune pathways implicated in the pathogenesis of HF and IBD, and that both conditions exhibit complex immune-fibrotic regulatory networks (**Figure 14**).

The NOD-like receptor (NLR) and Toll-like receptor (TLR) signaling pathways exhibited significant enrichment among common up-regulated genes. As members of pattern recognition receptors (PRRs), both NLRs and TLRs play critical roles in innate immune responses by recognizing danger signals. In the liver, Kupffer cells can sense stimuli from injured hepatocytes and microbial pathogens, producing IL-1β, IL-18, TNF-α, and various chemokines to recruit other immune cells, thereby promoting hepatic inflammatory cascades (19). In IBD, gut barrier dysfunction enables bacterial translocation, activating mononuclear phagocytes via PRRs. This triggers the release of IL-1β, TNF-α, IL-6, and IL-23, initiating downstream immune activation and perpetuating intestinal inflammation (21). Multiple studies have demonstrated that IL-23 blockade alleviates both Crohn’s disease and ulcerative colitis (22, 23). Our findings further underscore the importance of NLRs and TLRs in both diseases, implying aberrant overactivation of innate immune responses in their pathogenesis, which provides novel insights into the shared molecular mechanisms between these disorders.

Chemokines have a major influence on the chemotaxis and infiltration of immune cells. Our study confirmed that up-regulated genes are significantly enriched in “leukocyte chemotaxis” and “chemokine signaling pathway”, suggesting that targeting the chemokine signaling pathway would be effective for alleviating the diseases. Chemokines such as CXCL9, CXCL10, CXCL16, and CCL20 are involved in the recruitment of lymphocytes, while monocytes are recruited through CCL2 (19). Monocyte-derived macrophages play significant roles in liver inflammation. Notably, scar-associated macrophages(SAMs) can secrete TGF-β, IL-1β, and TNF-α to induce hepatic stellate cell activation, representing a critical cellular population driving fibrogenesis (24). Interfering with CCR2+ monocyte recruitment through pharmacological means effectively reduces hepatic inflammation and attenuates fibrosis (25). It has shown that CCR2+ monocytes can promote colonic fibrosis by producing TIMP-1, which inhibits collagen degradation (26). Neutrophil infiltration is mediated by CXCL1 and CXCL8 (27, 28), Neutrophils can secrete IL-17 to promote the activation of hepatic stellate cells in HF (19, 29), and in IBD, they can produce IL-1 and IL-22, exacerbating intestinal inflammatory responses (21). Our studies reveal shared genetic networks governing leukocyte migration and chemotaxis between HF and IBD, establishing a conceptual framework for future in-depth mechanistic investigations.

Cytokines such as IFN-γ, IL-17, and IL-22 secreted by lymphocytes also play crucial roles in the processes of inflammation and fibrosis. In our study, the IL-17 signaling and Th17 cell differentiation pathway were significantly enriched among the up-regulated genes, indicating Th17 responses as a shared pathogenic feature for both diseases. Evidence has verified that IL-1, IL-6, and IL-17 exert pro-inflammatory activities in HF and IBD, while IL-10 and IL-35 demonstrate anti-inflammatory activities (30, 31). A recent study reported that PBX1 promotes HF through IL-17 signaling transduction in hepatic stellate cells (32), and the development of liver fibrosis would be alleviated when blocking the IL-17 signaling axis (33, 34). The level of IL-17 is significantly elevated in the stricture areas of the intestine in Crohn’s disease (35), and IL-17 in the intestinal mucosa promotes fibrosis by facilitating epithelial-mesenchymal transition (36). The convergence of evidence reinforces our identification of IL-17 signaling as a shared pathogenic pathway, indicating its therapeutic potential in managing comorbid conditions. Moreover, IL-22 derived from Th17 cells demonstrates significant upregulation in HF, where it potentiates TGF-β signaling in hepatic stellate cells via the p38/MAPK pathway to accelerate fibrosis (29). In summary, our findings highlight similar cytokine regulatory networks underlying HF and IBD, providing critical insights for developing immunotherapeutic strategies to concurrently ameliorate both conditions.

To identify common biomarkers, we established a PPI network utilizing the intersected DEGs, and further utilized the MCC algorithm in Cytoscape software to select top 30 genes. Based on these 30 genes, we deployed two machine learning algorithms to determine feature genes of HF and IBD. The intersection yielded four genes MMP2, COL1A2, STAT1, and CXCL1, which are validated to have diagnostic significance for both diseases.

MMP2 (Matrix Metallopeptidase 2) is a zinc-dependent enzyme located on chromosome 16 and belongs to the Matrix Metalloproteinase (MMP) family. MMP2 exhibits the ability to degrade gelatin, as well as type IV collagen, which is an important element of the basement membrane in the ECM. Furthermore, MMP2 is critical for processes such as tissue repair and tumor invasion (37, 38). COL1A2 encodes one of the chains of type I collagen, along with two COL1A1 chains, forms the triple helix structure of type I collagen to be a crucial component of the ECM (39). Based on previous studies, both COL1A2 and MMP2 have been examined as fibrosis-related genes to assess tissue fibrosis status (40, 41). In single-cell sequencing data of HF and IBD, both MMP2 and COL1A2 are highly expressed in myofibroblasts, further underscoring the crucial role of myofibroblasts in promoting fibrosis. STAT1 is a member of the STAT protein family. Upon stimulation by cytokines or growth factors, STAT1 undergoes phosphorylation by JAKs to form dimers, which then translocate into the nucleus to modulate gene expression (42). Inhibition of STAT1 has been shown to mitigate inflammatory diseases mediated by Th1 and Th17 cells (43). In the context of colitis, STAT1 is implicated in mediating the pro-inflammatory effects of GBP5 (44), and the therapeutic efficacy of Tofacitinib in treating ulcerative colitis is associated with the downregulation of STAT1 expression (45). IFN-γ induces endothelial-mesenchymal transition by activating the JAK/STAT1 signaling pathway, thereby promoting hepatic fibrosis (46). However, it has also been reported that STAT1 activation in hepatic stellate cells (HSCs) triggers apoptosis, which exerts anti-fibrotic effects (47). These findings appear contradictory and necessitate further investigation. CXCL1 belongs to the CXC chemokine subfamily and has been shown to regulate cell chemotaxis, particularly of neutrophils (28, 48). Studies have shown that the administration of a high-fat diet alongside excessive alcohol consumption in mice results in the enhanced expression of CXCL1 in the liver and stimulates liver neutrophil infiltration, ultimately inducing acute liver inflammation and damage (49). CXCL1 can activate HSCs through autocrine mechanisms, thereby contributing to the progression of hepatic fibrosis (50). Other studies have identified a pivotal role for NOD2-NFκB/AP1-CXCL1/CXCL2 signaling pathway in the pathogenesis of IBD (51). Interestingly, The NOD-like receptor signaling pathway is significantly enriched among the up-regulated genes in our study. This report corroborates our findings and suggests that targeting the NOD2/CXCL1 signaling axis is a viable strategy for treating IBD. In summary, these four genes have distinct functional implications and have been described in the context of HF and IBD, but their specific mechanisms still require further investigation.

In this study, we have identified these four genes as shared biomarkers for HF and IBD from a cross-disease perspective for the first time. They exhibit consistently stable expression patterns across both training and validation sets for two diseases. The AUC values were all above 0.7 among ROC curves, with some even exceeding 0.9, indicating the reliable diagnostic predictive capability of these four genes. The successful construction of the logistic regression model further attests to the comprehensive diagnostic efficacy of four key genes. They also serve as potential therapeutic targets. These findings offer perspectives for novel diagnostic and therapeutic approaches for IBD and HF, especially when these two diseases coexist.

Notably, in single-cell sequencing of IBD, all four key genes are highly expressed in myofibroblasts, which further emphasizes the significant role of myofibroblasts in promoting intestinal fibrosis. Previous studies have reported that in IBD, activated mesenchymal cells not only produce ECM but also generate chemokines and cytokines, forming a positive feedback loop that promotes immune responses and fibrosis (52). However, in single-cell sequencing data of HF, the expression of CXCL1 and STAT1 is not prominent in myofibroblasts, reflecting functional heterogeneity of these cells across different tissues. In single-cell sequencing data of HF, CXCL1 and STAT1 are significantly highly expressed in cholangiocytes. A recent study reported that Osteopontin promotes the secretion of CCL2, CCL5, and CXCL1 by cholangiocytes, facilitating the aggregation of pro-inflammatory monocytes (53). This finding supports our results and further highlights the crucial role of cholangiocytes in secreting chemokines to promote inflammatory responses.

It confirmed a significant upregulation of M1 macrophages in both diseases via immune infiltration analysis, suggesting that targeting M1 macrophages would be a promising strategy for mitigating these conditions. M1 macrophages are known to produce high levels of pro-inflammatory cytokines, including IL-6, IL-1β, and TNF-α, which exacerbate IBD and liver injury (54). Studies have reported that decreasing M1 macrophage polarization and enhancing M2 macrophage polarization can downregulate pro-inflammatory cytokine levels and mitigate CCl4-induced hepatic fibrosis in mice (55). Recently, it has been reported that Artesunate alleviates ulcerative colitis in mice by enhancing M2/M1 ratio through the inhibition of endoplasmic reticulum stress (56). Another investigation revealed that the administration of bone marrow-derived M1 macrophages via the tail vein can facilitate the accumulation of Ly6C^lo^ macrophages to the fibrotic liver, which secret MMPs to promote collagen degradation, and also recruit NK cells to promote HSCs apoptosis, thereby alleviating hepatic fibrosis in mice (57). This strategy may also hold potential for the treatment of intestinal fibrosis in IBD, but further research is warranted. The four key diagnostic genes exhibit significant correlations with various immune cells, suggesting a close relationship between these genes and immune responses.

To achieve a more profound comprehension of the mechanisms governing these four key diagnostic genes in disease, we further constructed genes-TFs and genes-miRNAs regulatory networks. There are as many as 18 miRNAs that have common regulatory effects on the four genes, meriting further investigation. We observed that both GATA2 and FOXC1 are associated with three target genes within the regulatory network, implying their significant roles. GATA2 is capable of binding to the DNA sequence “GATA” through two zinc finger domains and reorganize the chromatin near the binding site to activate or repress gene expression. It can also interact with other transcription factors (58). As a part of the FOX transcription factor family, FOXC1 functions in embryonic development and tumor progression (59). Additionally, STAT1, can modulate the expression of COL1A2, and the mechanism underlying their interaction in disease contexts require further investigation. Studies on these transcription factors in both diseases remain limited, and their regulatory interactions with target genes necessitate additional experimental validation. Based on these four key genes, we predicted potential therapeutic drugs that may have therapeutic effects on both diseases through the DSigDB database, but further experimental and clinical validation is essential.

Our study is not devoid of limitations. First, the sample size analyzed herein is confined, necessitating the incorporation of additional samples in future endeavors to validate our results and enhance the credibility of the research. Second, our research did not involve the establishment of cell and animal models to investigate the functions of key genes, thereby constraining our comprehension of their profound underlying mechanisms of action. Notwithstanding these constraints, our investigation offers fresh insights into the shared mechanisms underlying these two diseases, setting a foundational groundwork for subsequent scholarly pursuits. The identification of four crucial diagnostic genes opens up new avenues for the diagnosis and therapeutic intervention of both diseases, particularly when they co-occur.

## 5 Conclusion

In conclusion, we identified four key diagnostic genes: MMP2, COL1A2, CXCL1, and STAT1, which can serve as shared biomarkers for IBD and HF. We simultaneously constructed a logistic regression model based on four key diagnostic genes with good diagnostic performance. We further determined the correlations of key diagnostic genes with immune cells, constructed regulatory networks of genes-miRNAs and genes-TFs, predicted associated drugs, and verified the expression and distribution of these four genes in single-cell sequencing data. We have also indicated the similarities between HF and IBD in terms of immunity, metabolism, and fibrosis, specifically including immune cell infiltration and chemotaxis, intercellular adhesion, cytokine regulation, metabolic processes, enzymatic activities, and extracellular matrix deposition.

## Data Availability

The datasets presented in this study can be found in online repositories. The names of the repository/repositories and accession number(s) can be found in the article/**Supplementary Material**.

## References

[1] Kisseleva T Brenner D. Molecular and cellular mechanisms of liver fibrosis and its regression. Nat Rev Gastroenterol Hepatol. (2021) 18:151–66. doi: 10.1038/s41575-020-00372-7 33128017

[2] Liu D Saikam V Skrada KA Merlin D Iyer SS. Inflammatory bowel disease biomarkers. Med Res Rev. (2022) 42:1856–87. doi: 10.1002/med.21893 PMC10321231 35603998

[3] Liu Z Huang Z Wang Y Xiong S Lin S He J. Intestinal strictures in crohn’s disease: an update from 2023. United Eur Gastroenterol J. (2024) 12:802–13. doi: 10.1002/ueg2.12568 PMC11250166 38546434

[4] Rodriguez-Duque JC Calleja JL Iruzubieta P Hernández-Conde M Rivas-Rivas C Vera MI. Increased risk of mafld and liver fibrosis in inflammatory bowel disease independent of classic metabolic risk factors. Clin Gastroenterol Hepatol. (2023) 21:406–14.e7. doi: 10.1016/j.cgh.2022.01.039 35124272

[5] Manns MP Bergquist A Karlsen TH Levy C Muir AJ Ponsioen C. Primary sclerosing cholangitis. Nat Rev Dis Primers. (2025) 11:17. doi: 10.1038/s41572-025-00600-x 40082445

[6] Axiaris G Zampeli E Michopoulos S Bamias G. Management of hepatitis B virus infection in patients with inflammatory bowel disease under immunosuppressive treatment. World J Gastroenterol. (2021) 27:3762–79. doi: 10.3748/wjg.v27.i25.3762 PMC8291024 34321842

[7] Lee JM Wei SC Lee KM Ye BD Mao R Kim HS. Clinical course of hepatitis B viral infection in patients undergoing anti-tumor necrosis factor Α Therapy for inflammatory bowel disease. Gut Liver. (2022) 16:396–403. doi: 10.5009/gnl210081 34593670 PMC9099383

[8] Taneja V Stein DJ Feuerstein JD. Impact of cirrhosis on outcomes in inflammatory bowel disease hospitalizations. J Clin Gastroenterol. (2022) 56:718–23. doi: 10.1097/mcg.0000000000001640 35152240

[9] Liguori A Zoncapè M Casazza G Easterbrook P Tsochatzis EA. Staging liver fibrosis and cirrhosis using non-invasive tests in people with chronic hepatitis B to inform who 2024 guidelines: A systematic review and meta-analysis. Lancet Gastroenterol Hepatol. (2025) 10(4):332–49. doi: 10.1016/s2468-1253(24)00437-0 39983746

[10] Pozowski P Bilski M Bedrylo M Sitny P Zaleska-Dorobisz U. Modern ultrasound techniques for diagnosing liver steatosis and fibrosis: A systematic review with a focus on biopsy comparison. World J Hepatol. (2025) 17:100033. doi: 10.4254/wjh.v17.i2.100033 40027573 PMC11866135

[11] Lu C Rosentreter R Parker CE Remillard J Wilson SR Baker ME. International expert guidance for defining and monitoring small bowel strictures in crohn’s disease on intestinal ultrasound: A consensus statement. Lancet Gastroenterol Hepatol. (2024) 9:1101–10. doi: 10.1016/s2468-1253(24)00265-6 39447590

[12] Dolinger M Torres J Vermeire S. Crohn’s disease. Lancet. (2024) 403:1177–91. doi: 10.1016/s0140-6736(23)02586-2 38437854

[13] Le Berre C Honap S Peyrin-Biroulet L. Ulcerative colitis. Lancet. (2023) 402:571–84. doi: 10.1016/s0140-6736(23)00966-2 37573077

[14] Lorenzo-Zúñiga V Bartolí R Planas R Hofmann AF Viñado B Hagey LR. Oral bile acids reduce bacterial overgrowth, bacterial translocation, and endotoxemia in cirrhotic rats. Hepatology. (2003) 37:551–7. doi: 10.1053/jhep.2003.50116 12601352

[15] Bajaj JS Heuman DM Hylemon PB Sanyal AJ White MB Monteith P. Altered profile of human gut microbiome is associated with cirrhosis and its complications. J Hepatol. (2014) 60:940–7. doi: 10.1016/j.jhep.2013.12.019 PMC3995845 24374295

[16] Leibovitzh H Nayeri S Borowski K Hernandez-Rocha C Lee SH Turpin W. Inflammatory bowel disease associated with primary sclerosing cholangitis is associated with an altered gut microbiome and bile acid profile. J Crohns Colitis. (2024) 18:1957–66. doi: 10.1093/ecco-jcc/jjae096 PMC11637524 38980940

[17] Bruneau A Hundertmark J Guillot A Tacke F. Molecular and cellular mediators of the gut-liver axis in the progression of liver diseases. Front Med (Lausanne). (2021) 8:725390. doi: 10.3389/fmed.2021.725390 34650994 PMC8505679

[18] Dorner H Stolzer I Mattner J Kaminski S Leistl S Edrich LM. Gut pathobiont-derived outer membrane vesicles drive liver inflammation and fibrosis in primary sclerosing cholangitis-associated inflammatory bowel disease. Gastroenterology. (2024) 167:1183–97.e16. doi: 10.1053/j.gastro.2024.06.032 38992449

[19] Hammerich L Tacke F. Hepatic inflammatory responses in liver fibrosis. Nat Rev Gastroenterol Hepatol. (2023) 20:633–46. doi: 10.1038/s41575-023-00807-x 37400694

[20] D’Alessio S Ungaro F Noviello D Lovisa S Peyrin-Biroulet L Danese S. Revisiting fibrosis in inflammatory bowel disease: the gut thickens. Nat Rev Gastroenterol Hepatol. (2022) 19:169–84. doi: 10.1038/s41575-021-00543-0 34876680

[21] Friedrich M Pohin M Powrie F. Cytokine networks in the pathophysiology of inflammatory bowel disease. Immunity. (2019) 50:992–1006. doi: 10.1016/j.immuni.2019.03.017 30995511

[22] Saab O Al-Obaidi H Algodi M Algodi A Rashid Y Al-Sagban A. Interlukin-23 inhibitors as an induction and maintenance therapy for moderate to severe ulcerative colitis: A systematic review and meta−Analysis of randomized controlled trials. Inflammation Res. (2025) 74:50. doi: 10.1007/s00011-025-02017-4 40057620

[23] Cappello M Celsa C. The vivid-1 study: A novel methodological approach provides further evidence of efficacy of selective il-23 inhibition in crohn’s disease. Med. (2025) 6:100572. doi: 10.1016/j.medj.2024.100572 39954670

[24] Horn P Tacke F. Metabolic reprogramming in liver fibrosis. Cell Metab. (2024) 36:1439–55. doi: 10.1016/j.cmet.2024.05.003 38823393

[25] Krenkel O Puengel T Govaere O Abdallah AT Mossanen JC Kohlhepp M. Therapeutic inhibition of inflammatory monocyte recruitment reduces steatohepatitis and liver fibrosis. Hepatology. (2018) 67:1270–83. doi: 10.1002/hep.29544 28940700

[26] Kuroda N Masuya M Tawara I Tsuboi J Yoneda M Nishikawa K. Infiltrating ccr2(+) monocytes and their progenies, fibrocytes, contribute to colon fibrosis by inhibiting collagen degradation through the production of timp-1. Sci Rep. (2019) 9:8568. doi: 10.1038/s41598-019-45012-6 31189971 PMC6562037

[27] Imada A Ina K Shimada M Yokoyama T Yokoyama Y Nishio Y. Coordinate upregulation of interleukin-8 and growth-related gene product-alpha is present in the colonic mucosa of inflammatory bowel. Scand J Gastroenterol. (2001) 36:854–64. doi: 10.1080/003655201750313397 11495082

[28] Girbl T Lenn T Perez L Rolas L Barkaway A Thiriot A. Distinct compartmentalization of the chemokines cxcl1 and cxcl2 and the atypical receptor ackr1 determine discrete stages of neutrophil diapedesis. Immunity. (2018) 49:1062–76.e6. doi: 10.1016/j.immuni.2018.09.018 30446388 PMC6303217

[29] Fabre T Molina MF Soucy G Goulet JP Willems B Villeneuve JP. Type 3 cytokines il-17a and il-22 drive tgf-Β-dependent liver fibrosis. Sci Immunol. (2018) 3(28):eaar7754. doi: 10.1126/sciimmunol.aar7754 30366940

[30] Zhang Z Wang J Li H Niu Q Tao Y Zhao X. The role of the interleukin family in liver fibrosis. Front Immunol. (2025) 16:1497095. doi: 10.3389/fimmu.2025.1497095 39995661 PMC11847652

[31] Rieder F Mukherjee PK Massey WJ Wang Y Fiocchi C. Fibrosis in ibd: from pathogenesis to therapeutic targets. Gut. (2024) 73:854–66. doi: 10.1136/gutjnl-2023-329963 PMC10997492 38233198

[32] Zhao Q Liu H Yang Z Han X Kang A Li H. Pre-B-cell leukemia transcription factor 1 contributes to liver fibrosis by enabling il-7 signaling in hepatic stellate cells. Hepatology. (2025). doi: 10.1097/hep.0000000000001302 40080840

[33] Dabbaghizadeh A Dion J Maali Y Fouda A Bédard N Evaristo G. Novel rorγt inverse agonists limit il-17-mediated liver inflammation and fibrosis. J Immunol. (2025). doi: 10.1093/jimmun/vkaf014 PMC12207071 40073158

[34] Jiang S Jiang Y Feng J Hou J Qin Z Wang Y. Triptolide combined with salvianolic acid B alleviates ccl(4)-induced liver fibrosis by suppressing the th17/il-17a axis. Int Immunopharmacol. (2025) 150:114300. doi: 10.1016/j.intimp.2025.114300 39965387

[35] Cho YB Park KJ Yoon SN Song KH Kim DS Jung SH. Long-term results of adipose-derived stem cell therapy for the treatment of crohn’s fistula. Stem Cells Transl Med. (2015) 4:532–7. doi: 10.5966/sctm.2014-0199 PMC4414218 25829404

[36] Panés J García-Olmo D Van Assche G Colombel JF Reinisch W Baumgart DC. Long-term efficacy and safety of stem cell therapy (Cx601) for complex perianal fistulas in patients with crohn’s disease. Gastroenterology. (2018) 154:1334–42.e4. doi: 10.1053/j.gastro.2017.12.020 29277560

[37] He L Kang Q Chan KI Zhang Y Zhong Z Tan W. The immunomodulatory role of matrix metalloproteinases in colitis-associated cancer. Front Immunol. (2023) 13:1093990. doi: 10.3389/fimmu.2022.1093990 36776395 PMC9910179

[38] Madzharova E Kastl P Sabino F Auf dem Keller U. Post-translational modification-dependent activity of matrix metalloproteinases. Int J Mol Sci. (2019) 20(12):3077. doi: 10.3390/ijms20123077 31238509 PMC6627178

[39] Gelse K. Collagens—Structure, function, and biosynthesis. Advanced Drug Delivery Rev. (2003) 55:1531–46. doi: 10.1016/j.addr.2003.08.002 14623400

[40] Prados ME García-Martín A Unciti-Broceta JD Palomares B Collado JA Minassi A. Betulinic acid hydroxamate prevents colonic inflammation and fibrosis in murine models of inflammatory bowel disease. Acta Pharmacologica Sin. (2020) 42:1124–38. doi: 10.1038/s41401-020-0497-0 PMC8209138 32811965

[41] Chale-Dzul J Pérez-Cabeza de Vaca R Quintal-Novelo C Olivera-Castillo L Moo-Puc R. Hepatoprotective effect of a fucoidan extract from sargassum fluitans borgesen against ccl(4)-induced toxicity in rats. Int J Biol Macromol. (2020) 145:500–9. doi: 10.1016/j.ijbiomac.2019.12.183 31874267

[42] Butturini E Carcereri de Prati A Mariotto S. Redox regulation of stat1 and stat3 signaling. Int J Mol Sci. (2020) 21(42):7034. doi: 10.3390/ijms21197034 32987855 PMC7582491

[43] Park J Son MJ Ho CC Lee SH Kim Y An J. Transcriptional inhibition of stat1 functions in the nucleus alleviates th1 and th17 cell-mediated inflammatory diseases. Front Immunol. (2022) 13:1054472. doi: 10.3389/fimmu.2022.1054472 36591260 PMC9800178

[44] Li Y Wang W Zhu R Zhu X Sun M Huang Y. Stat1 mediates the pro-inflammatory role of gbp5 in colitis. Commun Biol. (2025) 8:385. doi: 10.1038/s42003-025-07843-0 40055493 PMC11889220

[45] van Gennep S Fung ICN Jong DC Ramkisoen RK Clasquin E de Jong J. Histological outcomes and jak-stat signalling in ulcerative colitis patients treated with tofacitinib. J Crohns Colitis. (2024) 18:1283–91. doi: 10.1093/ecco-jcc/jjae031 PMC11324337 38506097

[46] Wang T Liu B Huang J Zhao Q Shen H Bi T. Ifn-Γ-mediated inhibition of jak/stat signaling via nano-scutellarin treatment is an efficient strategy for ameliorating liver fibrosis. J Transl Med. (2025) 23:195. doi: 10.1186/s12967-025-06155-5 39962553 PMC11834254

[47] Wang C Bai Y Li T Liu J Wang Y Ju S. Ginkgetin exhibits antifibrotic effects by inducing hepatic stellate cell apoptosis via stat1 activation. Phytother Res. (2024) 38:1367–80. doi: 10.1002/ptr.8106 38217097

[48] Korbecki J Barczak K Gutowska I Chlubek D Baranowska-Bosiacka I. Cxcl1: gene, promoter, regulation of expression, mrna stability, regulation of activity in the intercellular space. Int J Mol Sci. (2022) 23(2):792. doi: 10.3390/ijms23020792 35054978 PMC8776070

[49] Chang B Xu MJ Zhou Z Cai Y Li M Wang W. Short- or long-term high-fat diet feeding plus acute ethanol binge synergistically induce acute liver injury in mice: an important role for cxcl1. Hepatology. (2015) 62:1070–85. doi: 10.1002/hep.27921 PMC4589443 26033752

[50] Shi WP Ju D Li H Yuan L Cui J Luo D. Cd147 promotes cxcl1 expression and modulates liver fibrogenesis. Int J Mol Sci. (2018) 19(4):1145. doi: 10.3390/ijms19041145 29642635 PMC5979418

[51] Wang J Sun M Liu X Yan Q Gao Q Ni K. Transcriptome analysis identifies genetic risk markers and explores the pathogenesis for inflammatory bowel disease. Biochim Biophys Acta Mol Basis Dis. (2024) 1870:167013. doi: 10.1016/j.bbadis.2023.167013 38199515

[52] Kalafateli M Tourkochristou E Tsounis EP Aggeletopoulou I Triantos C. New insights into the pathogenesis of intestinal fibrosis in inflammatory bowel diseases: focusing on intestinal smooth muscle cells. Inflammation Bowel Dis. (2025) 31:579–92. doi: 10.1093/ibd/izae292 39680685

[53] Coombes JD Manka PP Swiderska-Syn M Vannan DT Riva A Claridge LC. Osteopontin promotes cholangiocyte secretion of chemokines to support macrophage recruitment and fibrosis in mash. Liver Int. (2025) 45:e16131. doi: 10.1111/liv.16131 39422353 PMC11893260

[54] Wang C Ma C Gong L Guo Y Fu K Zhang Y. Macrophage polarization and its role in liver disease. Front Immunol. (2021) 12:803037. doi: 10.3389/fimmu.2021.803037 34970275 PMC8712501

[55] Wu BM Liu JD Li YH Li J. Margatoxin mitigates ccl4−Induced hepatic fibrosis in mice via macrophage polarization, cytokine secretion and stat signaling. Int J Mol Med. (2020) 45:103–14. doi: 10.3892/ijmm.2019.4395 PMC6889929 31746414

[56] Yin S Li L Chen X Wang J Mao Y Wang J. Artesunate alleviated murine ulcerative colitis by regulating immune response through inhibiting endoplasmic reticulum stress. Front Immunol. (2025) 16:1545468. doi: 10.3389/fimmu.2025.1545468 40079012 PMC11897576

[57] Ma PF Gao CC Yi J Zhao JL Liang SQ Zhao Y. Cytotherapy with M1-polarized macrophages ameliorates liver fibrosis by modulating immune microenvironment in mice. J Hepatol. (2017) 67:770–9. doi: 10.1016/j.jhep.2017.05.022 28596109

[58] Aktar A Heit B. Role of the pioneer transcription factor gata2 in health and disease. J Mol Med (Berl). (2023) 101:1191–208. doi: 10.1007/s00109-023-02359-8 37624387

[59] Han B Bhowmick N Qu Y Chung S Giuliano AE Cui X. Foxc1: an emerging marker and therapeutic target for cancer. Oncogene. (2017) 36:3957–63. doi: 10.1038/onc.2017.48 PMC5652000 28288141

