# Supplementary figures and images for: Exploring shared pathogenic mechanisms and biomarkers in hepatic fibrosis and inflammatory bowel disease through bioinformatics and machine learning
Source: Front Immunol. 2025 May 12;16:1533246. doi: 10.3389/fimmu.2025.1533246 (PMC12104268; doi:10.3389/fimmu.2025.1533246)

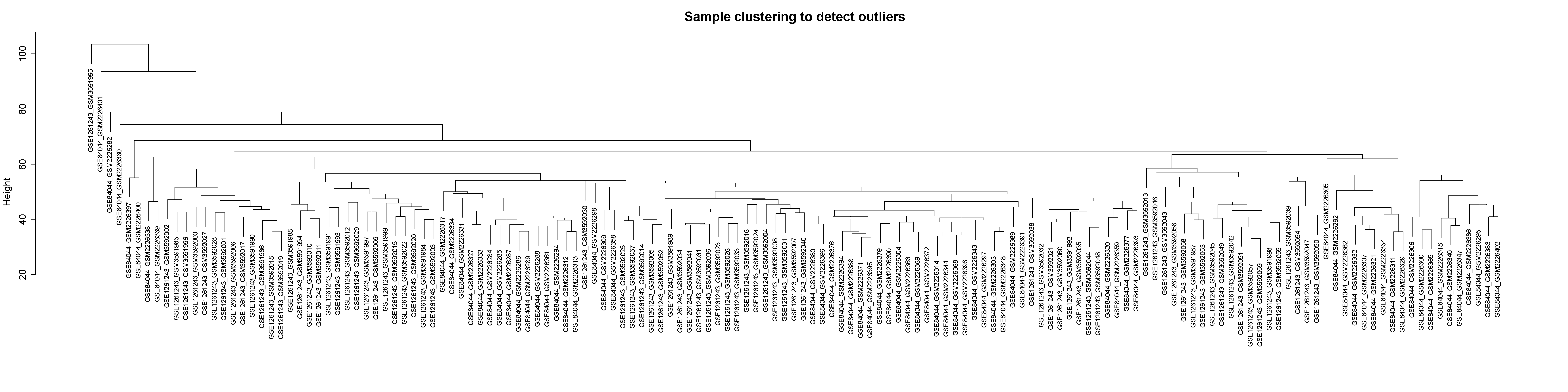

Supplement: Supplementary file 1 [file Image1.tif]

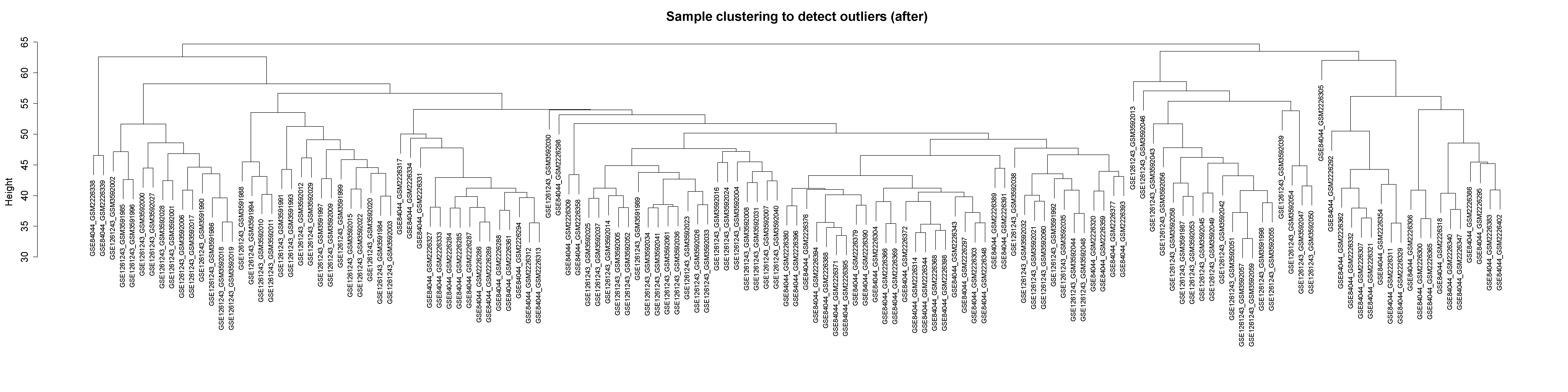

Supplement: Supplementary file 2 [file Image2.tif]

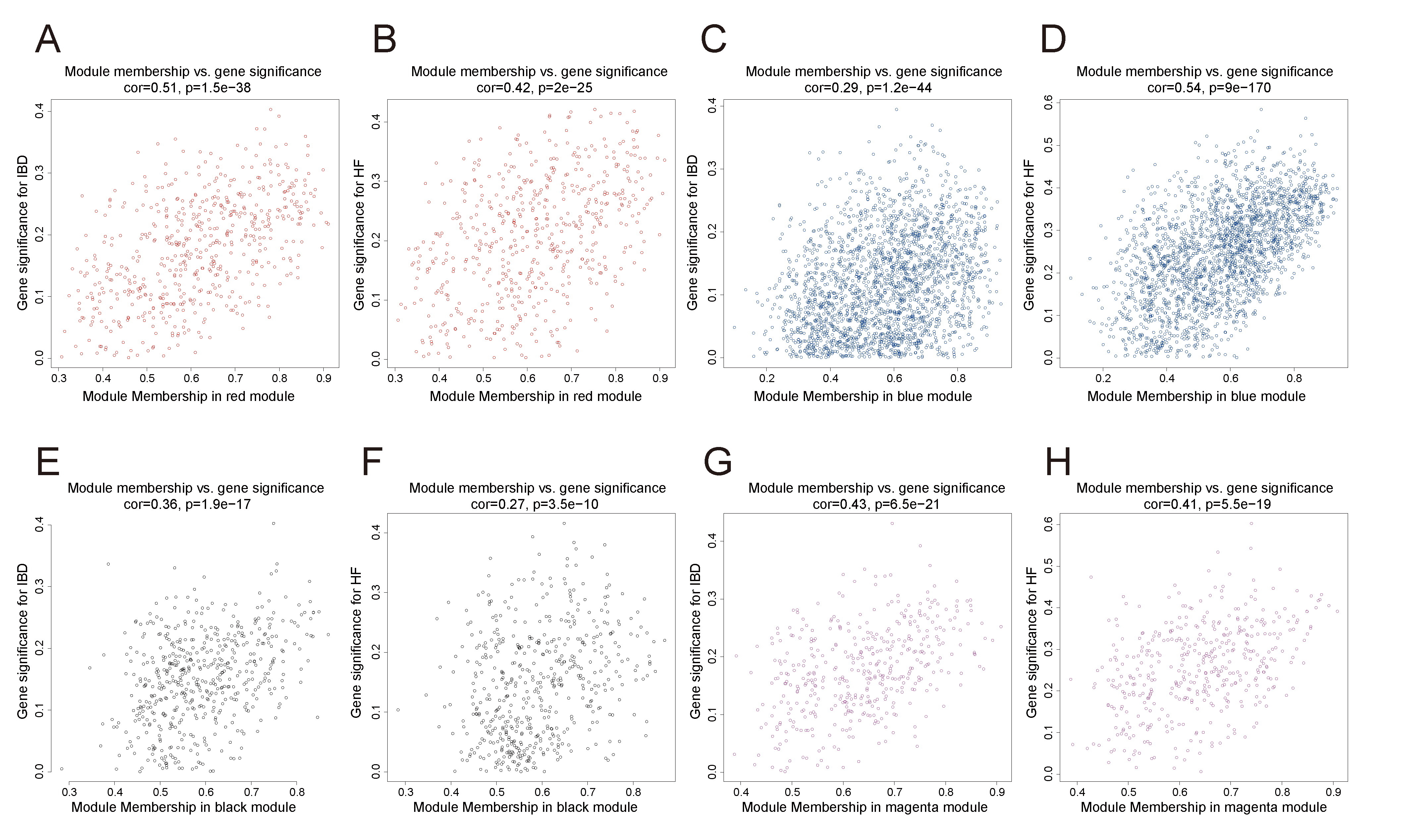

Supplement: Supplementary file 3 [file Image3.tif]

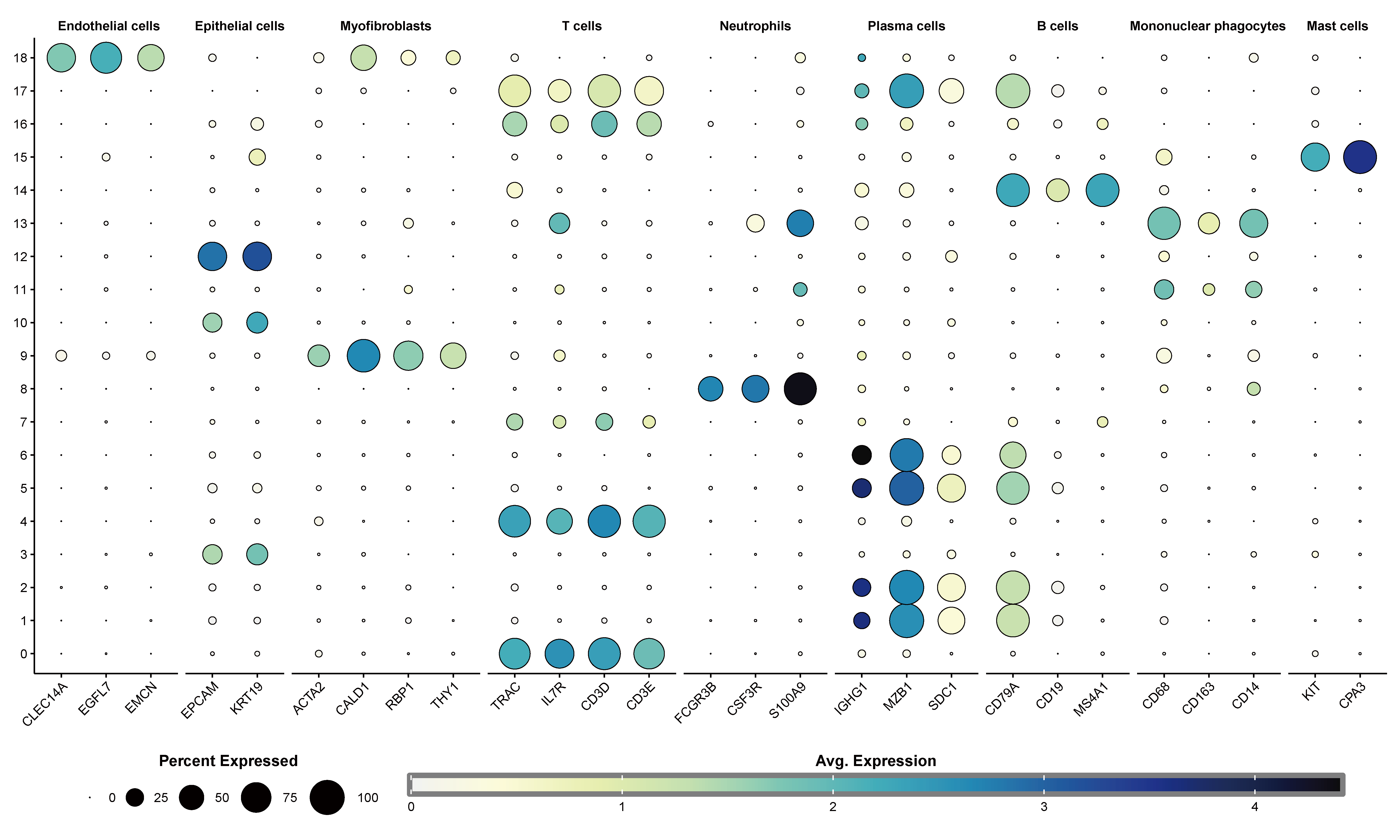

Supplement: Supplementary file 4 [file Image4.tif]

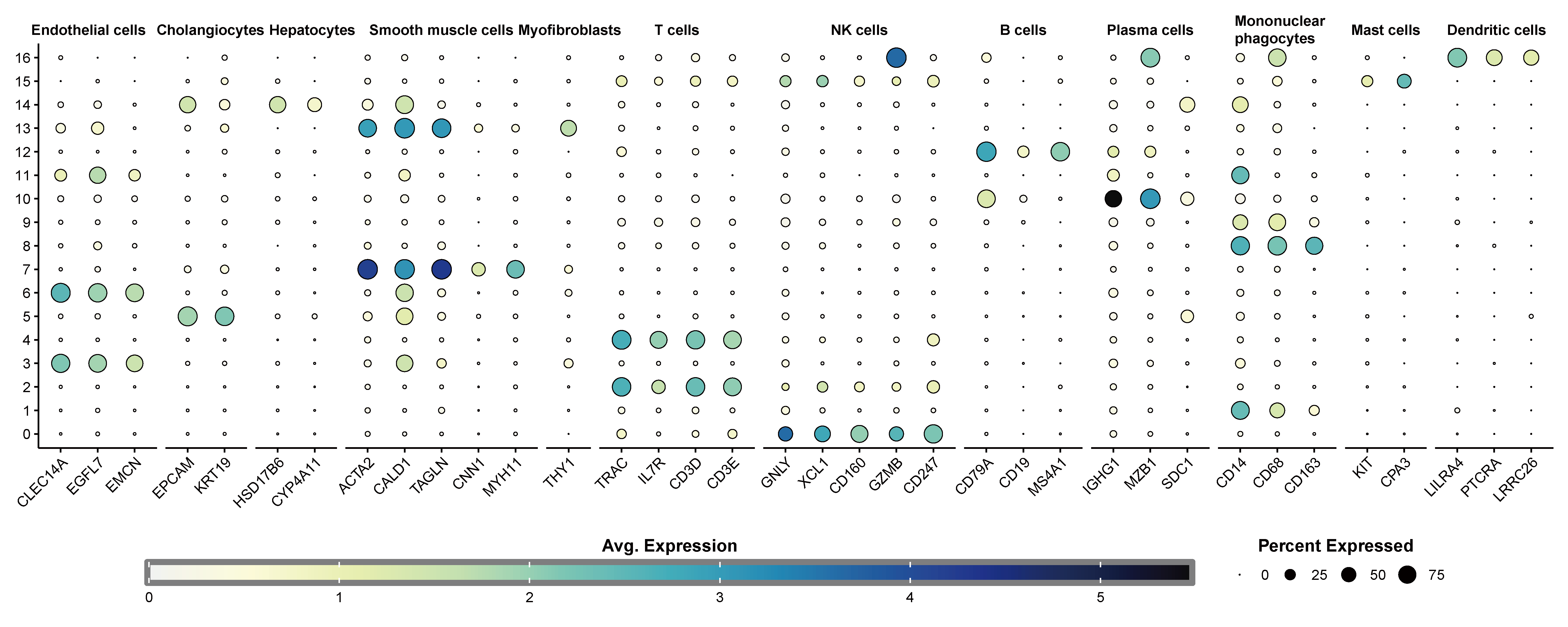

Supplement: Supplementary file 5 [file Image5.tif]
